# Predicted Functions of MdmX in Fine-Tuning the Response of p53 to DNA Damage

**DOI:** 10.1371/journal.pcbi.1000665

**Published:** 2010-02-05

**Authors:** Sohyoung Kim, Mirit I. Aladjem, Geoffrey B. McFadden, Kurt W. Kohn

**Affiliations:** 1 Laboratory of Molecular Pharmacology, National Cancer Institute, National Institute of Health, Bethesda, Maryland, United States of America; 2 Mathematical and Computational Sciences Division, Information Technology Laboratory, National Institute of Standards and Technology, Gaithersburg, Maryland, United States of America; Sony Computer Science Laboratories Inc., Systems Biology Institute, Japan

## Abstract

Tumor suppressor protein p53 is regulated by two structurally homologous proteins, Mdm2 and MdmX. In contrast to Mdm2, MdmX lacks ubiquitin ligase activity. Although the essential interactions of MdmX are known, it is not clear how they function to regulate p53. The regulation of tumor suppressor p53 by Mdm2 and MdmX in response to DNA damage was investigated by mathematical modeling of a simplified network. The simplified network model was derived from a detailed molecular interaction map (MIM) that exhibited four coherent DNA damage response pathways. The results suggest that MdmX may amplify or stabilize DNA damage-induced p53 responses via non-enzymatic interactions. Transient effects of MdmX are mediated by reservoirs of p53∶MdmX and Mdm2∶MdmX heterodimers, with MdmX buffering the concentrations of p53 and/or Mdm2. A survey of kinetic parameter space disclosed regions of switch-like behavior stemming from such reservoir-based transients. During an early response to DNA damage, MdmX positively or negatively regulated p53 activity, depending on the level of Mdm2; this led to amplification of p53 activity and switch-like response. During a late response to DNA damage, MdmX could dampen oscillations of p53 activity. A possible role of MdmX may be to dampen such oscillations that otherwise could produce erratic cell behavior. Our study suggests how MdmX may participate in the response of p53 to DNA damage either by increasing dependency of p53 on Mdm2 or by dampening oscillations of p53 activity and presents a model for experimental investigation.

## Author Summary

A Molecular Interaction Map (MIM) akin to a circuit diagram of an electric device can give a comprehensive view of cellular processes and help understand complex protein functions in cells. To this end, we generated a MIM focused on the p53-Mdm2-MdmX network proteins and performed computer simulations to help understand how Mdm2 and MdmX may regulate p53. Proper regulation of p53 is important for cell survival: elevated levels of p53 can lead to cell death, and decreased levels of p53 can lead to cancer. Mdm2 and MdmX are structurally homologous proteins that regulate p53. Mdm2 negatively regulates p53 by degradation, but MdmX regulation of p53 is not well understood. Recently, Mdm2 and MdmX have been recognized as potential cancer therapeutic targets. In an effort to better understand how MdmX can alter the p53 activity under various conditions, we used mathematical models based on the MIM network to generate hypotheses that can be tested by experiments. Our simulations suggest that MdmX may increase the dependency of p53 on Mdm2 or dampen p53 oscillations during DNA damage response.

## Introduction

The transcription factor p53 is a tumor suppressor that causes cell cycle arrest or apoptosis in response to stress signals [1]. Loss of p53 function by mutation or by disregulation often leads to cancer [1]. Excessive p53 protein results in premature aging [2] and cell death [1]. Thus, maintaining appropriate levels of p53 is essential for cell survival. Mdm2 and MdmX are structurally-related p53-binding proteins that play a key role in regulating the level of p53 [3]. Although several models of the p53-Mdm2 network have been proposed [4]–[14] and reproduced experimentally observed oscillatory or pulsatile behaviors, those models did not include MdmX and did not address potential impacts of MdmX on dynamics of the p53-Mdm2 network. MdmX can interfere with p53-Mdm2 negative feedback loop by interacting with those two molecules. As indicated by diverse effects of MdmX in biological experiments [15]–[18], it is not intuitively obvious what the consequence of that interference would be. In this work, we examined mathematical network models to address the role of MdmX in regulating the dynamics of p53 activity in relation to Mdm2.

Mdm2 is a ubiquitin ligase that negatively regulates p53 by promoting ubiqutin-dependent p53 degradation [3]. In response to DNA damage, Mdm2 is transcriptionally induced by p53 generating a delayed negative feedback loop between p53 and Mdm2 [19]. Mdm2-null mice die during the embryonic stages due to apoptosis induced by elevated p53 activity [19]. MdmX is structurally similar to Mdm2 and interacts with p53, but MdmX lacks ubiquitin ligase activity [20]. However, MdmX is critical for negative regulation of p53 as indicated by p53-dependent embryonic death in MdmX-null mice [21]–[23]. MdmX can bind Mdm2, but the effects of the ubiquitin ligase activity of Mdm2∶MdmX heterodimer on p53, if any, is not well understood. The role of MdmX in p53 regulation has been suggested to be either (1) a negative regulator, functioning as Mdm2 cofactor to enhance Mdm2-dependent p53 degradation [15], (2) a stabilizer that increases the level of p53 [16],[18], or (3) a positive regulator of p53 activity under stress condition [17].

In our model, MdmX is a key component. MdmX interacts with p53 or Mdm2 to form transcriptionally inactive p53∶MdmX or enzymatically inactive Mdm2∶MdmX. In order to model the interactions of MdmX with Mdm2 and p53, we selected a system of elementary processes that focused on the non-enzymatic interactions between MdmX and other molecules. Since the values of many kinetic constants for our model are unknown, we selected initial kinetic parameter sets to explore potential interesting behaviors. Then, we searched for regions of parameter space that showed biologically interesting behaviors and reproduced previously observed dynamic behaviors, including DNA damage induced oscillations [9] as well as the effects of Nutlin-induced inhibition of p53-Mdm2 binding [24]. Simulations of the model showed that simple binding interactions with p53 or Mdm2 by MdmX generated remarkably complex effects on the dynamics of the p53-Mdm2-MdmX network, such as amplification of p53 activity and damping of p53 oscillations.

## Results

### Heuristic Molecular Interaction Map (heuristic MIM)

**Figure 1:**
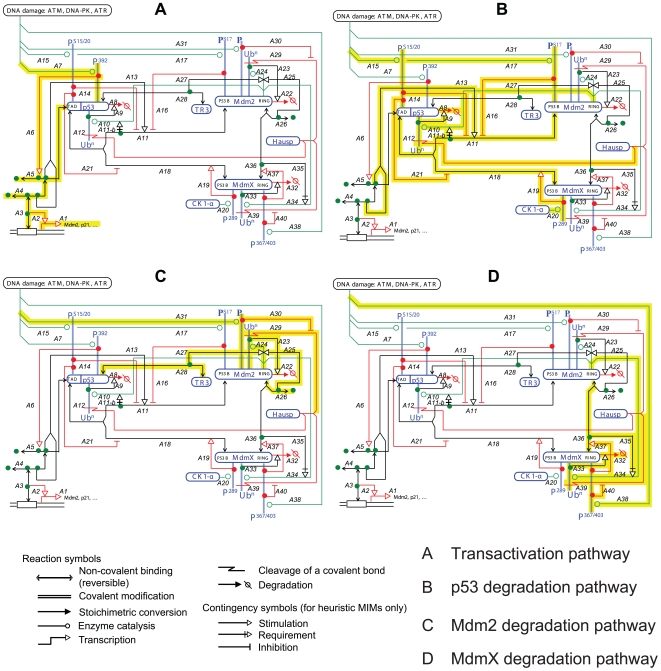
The p53-Mdm2-MdmX network displayed as a heuristic Molecular Interaction Map (MIM) [25]–[27]. Four pathways of p53 regulation are highlighted in the following panels: A) p53 transactivation, B) p53 degradation, C) Mdm2 degradation, D) MdmX degradation.

**Figure 2:**
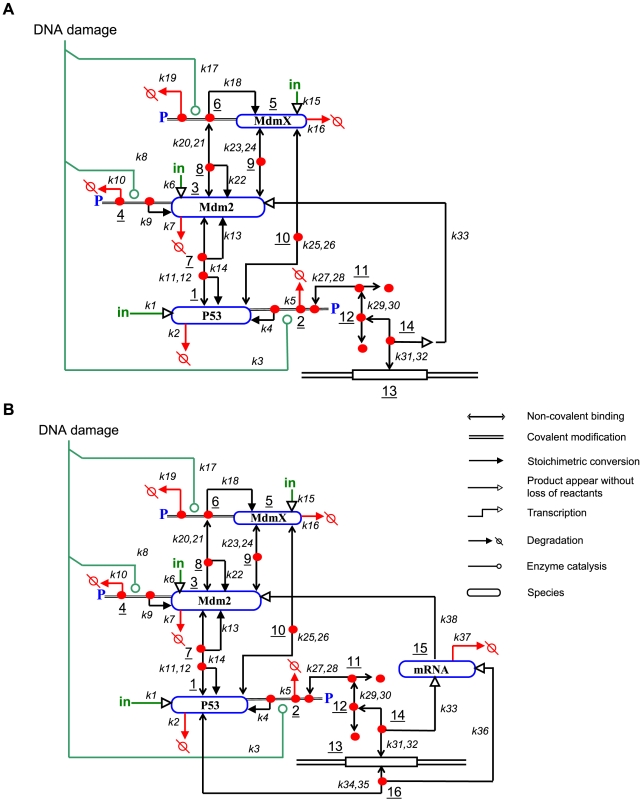
Network models, derived from the heuristic MIM shown in Figure 1, for simulation. Expressed here as simple (A) and full (B) explicit MIMs (see Kohn et al., 2006 for description of heuristic and explicit MIMs). The simple and full versions differ with respect to p53 basal production rate (k34, k35, k36) and mRNA (species 15): the simple network model does not include p53 basal production rate and mRNA species (in that case, species 14 directly generates Mdm2, species 3). Figure 3, 4, 5, 6, 7, 11 were based on the simple model (A), and Figure 8, 9, 10 were based on the full model (B). Definition of symbols can be found in Table 1 and Table S2.

To lay the foundation of a model, we first assembled the known molecular interactions among p53, Mdm2, and MdmX in the form of a heuristic MIM using the previously described notation [25]–[27] (Figure 1). The heuristic MIM organizes information from which an explicit model (Figure 2) for simulations was extracted and portrayed as an explicit MIM (Kohn 2001). To facilitate understanding, the model was also represented as an informal diagram (Figure S1) [28]. Additionally, SBML model was provided in the Supporting Information (Text S8).

Four pathways of p53 regulation, functioning simultaneously, are highlighted in Figure 1 and summarized as follows. (Details and references for each interaction are provided in an annotation Table S1. The heuristic map with annotations can be found at http://discover.nci.nih.gov/mim)

#### Transactivation pathway (highlighted in Figure 1-A)

P53 activates (A3, A2) the transcription of target genes including Mdm2 and p21 (A1). Phosphorylation (A7, A6) and oligomerization (A5, A4) of p53 enhance the transactivation function (A3).

#### P53 degradation module (highlighted in Figure 1-B)

p53 ubiquitination (A10) is mediated by the ring domain of p53-bound Mdm2 (A11, A11-b), and the ubiquitinated p53 undergoes degradation (A9, A8). Oligomerized p53 is more efficiently ubiquitinated and degraded (A13) than monomeric p53 because oligomerization of p53 enhances p53-Mdm2 binding. Ubiquitinated p53 can be deubiquitinated by Hausp (A12). Mdm2 binds to the transactivation domain (TAD) of p53 (A11), and MdmX competitively binds to the same site of p53 (A18). Binding between p53 and MdmX is stimulated by MdmX phosphorylation, mediated by Casein Kinase 1-alpha (CK1-alpha) (A20, A19). It is not known whether CK1-alpha is regulated by DNA damage. DNA damage-induced phosphorylations of p53 (A15) and Mdm2 (A17) coherently inhibit (A14, A16, respectively) the formation of p53∶Mdm2 heterodimer, thereby allowing the active forms of p53 to accumulate. P53 Ser20 phosphorylation attenuates the binding between p53 and MdmX (A21).

#### Mdm2 degradation pathway (highlighted in Figure 1-C)

Mdm2 is auto-ubiquitinated by the Mdm2 ring domain (A24, A25, A26), and the ubiquitinated Mdm2 undergoes degradation (A22, A23). Mdm2 may have to form a homodimer to autoubiquitinate (A26), but it is not clear whether the autoubiquitination is in cis or trans. The Mdm2 autoubiqutination is enhanced (A27) by an interaction between an orphan receptor, TR3, and p53 (A28). Ubiquitinated Mdm2 can be deubiquitinated by Hausp (A29), and the deubiquitination is inhibited (A30) by DNA damage induced phosphorylation of Mdm2 at an unknown site (A31).

#### MdmX degradation pathway (highlighted in Figure 1-D)

The ring domains of MdmX and Mdm2 can bind each other to form an oligomer (A36), and MdmX is ubiquitinated by the ring domain of bound Mdm2 (A34). Ubiquitinated MdmX undergoes degradation (A33, A32). Ubiquitinated MdmX can be deubiquitinated by Hausp (A39). DNA damage induced phosphorylation controls the level of MdmX coherently via several pathways. DNA damage-induced MdmX phosphorylation (A38) enhances (A37) the binding between Mdm2 and MdmX (A36) which can result in the increased ubiquitination of MdmX (A35). MdmX phosphorylation (A38) inhibits (A40) the deubiquitination (A39) of MdmX by Hausp, which in turn increases ubiquitinated MdmX showing coherent regulation by DNA damage-induced kinases.

### Simulations with a simple model and initial parameter sets

#### MdmX can induce switch-like behaviors

**Figure 3:**
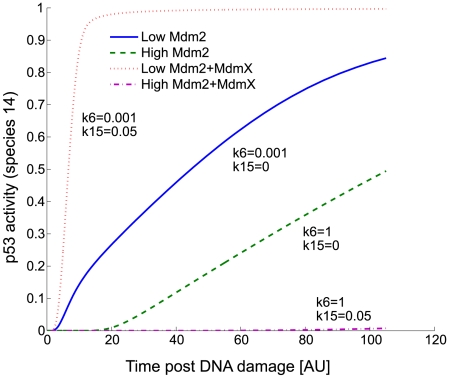
Simulated time series plot of the early DNA damage response. These simulations were based on the model in Figure 2-A with k33 = k14 = 0 because these reactions were considered not to play a significant role at early times. The system was equilibrated to steady state prior to DNA damage. Diverse transient dynamic behaviors of p53 activity (estimated by measuring occupancy of target promoters by p53 tetramers, species 14) were observed over a range of values of the basal production rate constant for Mdm2 (k6) and MdmX (k15).

Model simulations, based on the explicit MIM presented in Figure 2-A, were initially performed at various production rates of MdmX and Mdm2, and various levels of DNA damage (represented by the rate constants k3 = k8 = k17). P53 activity (species 14) was measured as output. For these initial simulations, a set of kinetic constants shown in Table 1 (column 4) was used. After pre-equilibration from the initial condition (Table S2), DNA damage was represented by various levels of phosphorylation rate constants (k3, k8, k17) and simulated during a short period of time (t = 0 to 55 (AU) post DNA damage). In these simulations, the p53-Mdm2 feedback loop was not included (k33 = 0), so as to focus on the early response of the system; that simplified the model and allowed us to understand the primary effects of various conditions on dynamics in the absence of the feedback loop. Examples of the transient dynamic behavior observed, when the production rates of Mdm2 and MdmX were varied, are shown in Figure 3. As one can note in Figure 3, p53 activity rises from zero because we assumed that there is no basal p53 activity in the absence of DNA damage in these initial simulations with simple network model (Figure 2-A).

**Table 1:** Kinetic constants used for simulations.

column # 1	column # 2	column # 3	column # 4	column # 5	column # 6	column # 7	column # 8	column # 9
Reaction #	Reactions (kinetic constant symbol)	Kinetic constant description	Initial parameter set (Fig 3,4,5,11)	osc param^a^ (Fig 6,7)	osc param^a^ (Fig S4)	osc param^a^ (Fig S3)	osc param^a^ (Fig 9A,10, S5A,S6,S8)	osc param^a^ (Fig 9B, S5B,S9)
1	In → x1 (k_1_)	Basal production of p53 (x1)	5.000E-02	Range^b^	1.000E-01	5.000E-01	0.003, 0.01^c^	0.003, 0.01 c
2	x1 → Ø (k_2_)	Basal degradation of p53 (x1)	2.100E-02	8.000E-04	4.400E-03	8.000E-04	3.923E-03	3.710E-03
3	x1 → x2 (k_3_)	Phosphorylation of p53 (x1)	Range^d^	Range^e^	1.000E+00	1.000E-01	1.578E-02	1.048E+01
4	x2 → x1 (k_4_)	Dephosphorylation of p53P (x2)	2.000E-02	2.365E-02	2.500E-01	2.365E-02	3.156E-04	2.095E-01
5	x2 → Ø (k_5_)	Degradation of p53P (x2)	1.700E-03	2.000E-03	4.400E-03	2.000E-03	1.224E-02	1.542E-03
6	In → x3 (k_6_)	Basal production of Mdm2 (x3)	Range^f^	1.500E-03	3.000E-03	1.500E-03	7.994E-05	6.528E-04
7	x3 → Ø (k_7_)	Basal degradation of Mdm2 (x3)	5.000E-03	5.000E-04	2.190E-02	5.000E-04	7.592E-05	1.275E-02
8	x3 → x4 (k_8_)	Phosphorylation of Mdm2 (x3)	Range^d^	2.000E-01	1.000E+00	2.000E-01	1.578E-02	1.048E+01
9	x4 → x3 (k_9_)	Dephosphorylation of Mdm2P (x4)	2.000E-02	2.000E-02	2.500E-01	2.000E-02	3.156E-04	2.095E-01
10	x4 → Ø (k_10_)	Degradation of Mdm2P (x4)	2.198E+01	5.000E-05	8.000E+00	5.000E-05	1.826E-01	2.436E-02
11	x1+x3 → x7 (k_11_)	Association between p53 (x1) and Mdm2 (x3)	1.000E-01	1.000E+00	1.000E+00	5.000E-01	3.056E+01	2.760E+00
12	x7 → x1+x3 (k_12_)	Dissociation of p53∶Mdm2 (x7)	5.000E-02	1.750E-02	1.000E-01	1.750E-02	1.608E-01	4.284E-01
13	x7 → x3 (k_13_)	Mdm2 dependent degradation of p53 (x1)	5.000E+00	2.500E+00	5.000E+00	1.000E+00	7.917E-01	9.259E+00
14	x7 → x1 (k_14_)	p53 dependent degradation of Mdm2 (x3)	0.000E+00	1.000E-01	0.000E+00	1.000E-01	1.583E-01	1.852E+00
15	In → x5 (k_15_)	Basal production of MdmX (x5)	Range^g^	0.000E+00	1.000E+00	Range^h^	2.173E-05	8.436E-05
16	x5 → Ø (k_16_)	Basal degradation of MdmX (x5)	5.000E-03	3.425E-02	6.850E-02	3.425E-02	3.155E-04	1.373E-04
17	x5 → x6 (k_17_)	Phosphorylation of MdmX (x5)	Range^d^	2.000E-02	1.000E+00	2.000E-02	1.578E-02	1.048E+01
18	x6 → x5 (k_18_)	Dephosphorylation of MdmXP (x6)	2.000E-02	2.000E-02	2.500E-01	2.000E-02	3.156E-04	2.095E-01
19	x6 → Ø (k_19_)	Degradation of MdmXP (x6)	5.000E-03	3.425E-02	6.850E-02	3.425E-02	1.224E-03	1.542E-04
20	x3+x6 → x8 (k_20_)	Association between Mdm2 (x3) and MdmXP (x6)	1.100E+01	4.150E-03	2.000E+00	4.150E-03	3.056E+01	2.760E+00
21	x8 → x3+x6 (k_21_)	Dissociation of Mdm2∶MdmXP (x8)	3.640E-05	8.000E-04	2.000E-03	8.000E-04	1.608E-01	4.284E-01
22	x8 → x3 (k_22_)	Mdm2 (x3) dependent degradation of MdmXP	7.319E+00	4.825E-02	2.000E+00	4.825E-02	7.917E-01	9.259E+00
23	x3+x5 → x9 (k_23_)	Association between Mdm2 (x3) and MdmX (x5)	1.000E-01	2.500E-01	9.372E-01	0.1211, Range^i^	3.427E+02	7.356E+00
24	x9 → x3+x5 (k_24_)	Dissociation of Mdm2∶MdmX (x9)	5.000E-02	3.500E-01	2.130E-02	1.830E-02	1.608E-01	4.284E-01
25	x1+x5 → x10 (k_25_)	Association between p53 (x1) and MdmX (x5)	1.000E-01	1.211E-01	3.925E-01	0.1211, Range^i^	2.596E-01	1.022E+01
26	x10 → x1+x5 (k_26_)	Dissociation of p53∶MdmX (x10)	5.000E-02	1.830E-02	5.000E-02	1.830E-02	1.608E-01	4.284E-01
27	x2+x2 → x11 (k_27_)	Dimerization of p53P (x2)	8.090E-01	5.000E-04	1.000E+00	5.000E-04	4.206E-01	2.535E-03
28	x11 → x2+x2 (k_28_)	Dissociation of p53P dimer, p53P∶p53P (x11)	6.110E-02	5.000E-04	5.000E-01	5.000E-04	7.918E-01	5.194E-02
29	x11+x11 → x12 (k_29_)	Dimerization of p53P∶p53P (x11)	8.090E-01	2.500E-05	1.000E+00	2.500E-05	1.543E+00	5.275E-02
30	x12 → x11+x11 (k_30_)	Dissociation of p53P tetramer, (p53P∶p53P): (p53P∶p53P) (x12)	6.110E-02	5.000E-04	5.000E-01	5.000E-04	1.016E-01	3.103E-01
31	x12+x13 → x14 (k_31_)	Association between p53P tetramer (x12) and promoter (x13)	8.090E-01	2.500E-05	1.000E+00	2.500E-05	4.715E-01	1.626E+00
32	x14 → x12+x13 (k_32_)	Dissociation of p53P tetramer and promoter complex (x14)	6.110E-02	5.000E-04	5.000E-01	5.000E-04	2.555E-01	3.059E-01
33	x14 → x3+x14 (k_33_ j)	Production of Mdm2 by p53P tetramer and promoter complex (x14)	0	1.000E-01	4.00E+01^k^	5.000E-01	NA	NA
34	x14 → x14+x15 (k_33_ l)	Production rate constant of mRNA	0	0	0	0	7.170E-01	1.801E+00
35	x15 → Ø (k_37_)	Degradation rate constant of mRNA	0	0	0	0	5.551E-02	3.813E-02
36	X15 → x3+x15 (k_38_)	Production rate constant of Mdm2 from mRNA	NA	NA	NA	NA	3.708E+00	1.456E+02
37	x1+x13 → x16 (k_34_)	Association rate constant between p53 and promoter	0	0	0	0	6.293E-03	4.015E-02
38	x16 → x1+x13 (k_35_)	Dissociation rate constant between p53 and promoter	0	0	0	0	2.794E+00	1.117E+02
39	x16 → x15+x16 (k_36_)	Basal transcription rate constant by p53 bound promoter	0	0	0	0	6.545E-02	9.043E-02

a Oscillatory condition.

b 26 logarithmically equally spaced points between 0.028118 and 10 ^1.

c 0.003 was used for the basal production rate of p53 in the absence of DNA damage. 0.01 was used for the basal production rate of p53 in the presence of DNA damage because the rate of p53 translation is increased after DNA damage. (Takagi et al., 2005).

d 50 logarithmically equally spaced points between 10̂-3 and 1.

e 50 logarithmically equally spaced points between 10̂-5 and 10̂1.

f 40 logarithmically equally spaced points between 10̂-3 and 10̂0.7.

g [0 0.05 5]

h [0.0001, 0.001, 0.005, 0.01, 0.02, 0.05, 0.075, 0.1, 0.5, 1, 2, 8, 10, 12, 20]

i 10 logarithmically equally spaced points between 10̂-5 and 20.0.

j k33 in figure 2-A.

k The reaction was used with time delay, 50.

l k33 in figure 2-B.

NA indicates that the interaction is not included.

**Figure 4:**
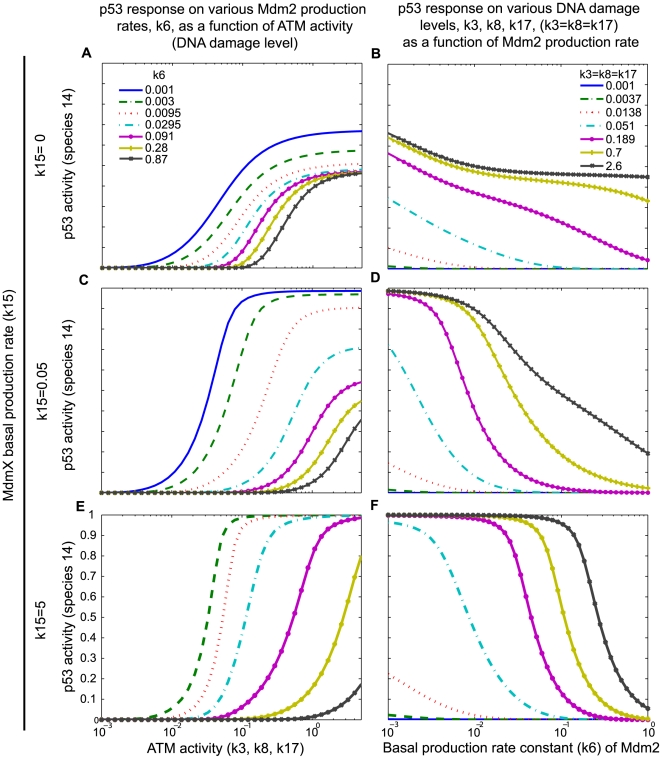
Dependence of p53 activity on DNA damage and Mdm2. Simulations were performed based on the model in Figure 2-A. Dependence of p53 activity on DNA damage-induced kinase activity (A, C, E) and Mdm2 basal production rate (B, D, F) during the early response to DNA damage (k33 = k14 = 0) is shown. The system was equilibrated to steady state prior to DNA damage. p53 activity was estimated by measuring occupancy of target promoters by p53 tetramers (species 14). Response curves are shown for three different production rate constants of MdmX (A, B: k15 = 0, C, D: k15 = 0.05, E, F: k15 = 5). In A, C, and E, the value of the Mdm2 basal production rate constant (k6) is indicated. In B, D, and F, the value of the DNA damage-induced phosphorylation rates (k3 = k8 = k17) is indicated.

In further simulations, an increased basal production rate constant (k6) of Mdm2 resulted in a decrease in p53 activity (Figure 4-A, C, E): negative regulation of p53 by Mdm2 was consistently observed with three different basal production rate constants of MdmX (k15) (Figure 4-B, D, F). In the absence of MdmX, p53 activity was attenuated by Mdm2 (Figure 4-A, B). Interestingly, MdmX increased the Mdm2-dependence of the p53 response to DNA damage (Figure 4-C, D, E, F), and generated switch-like Mdm2-dependence of p53 activity (Figure 4). This suggests that MdmX may confer onto the early p53 response a switch-like dependence on the basal production rate of Mdm2.

#### p53∶MdmX and Mdm2∶MdmX heterodimers act as reservoirs and generate switch-like p53 responses

**Figure 5:**
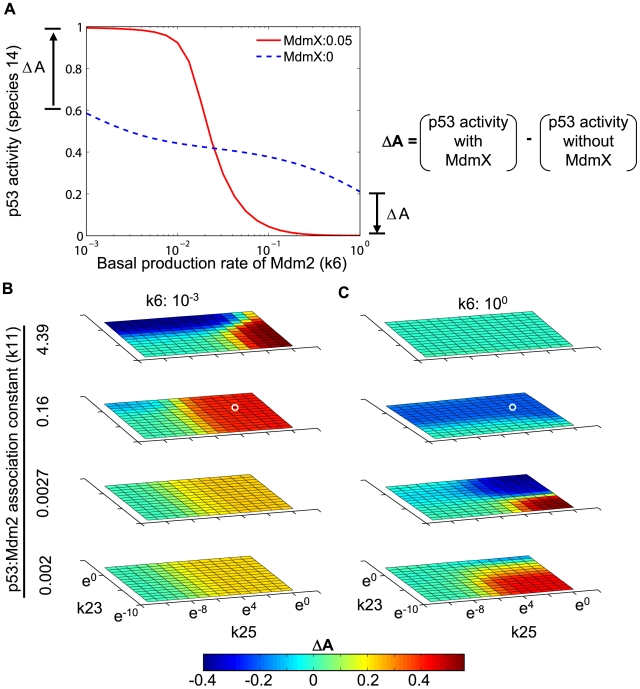
p53:MdmX and Mdm2:MdmX heterodimers act as reservoirs and generate switch-like dependence of p53. Simulations were done based on the model in Figure 2-A and done with k33 = k14 = 0. The system was equilibrated to steady state prior to DNA damage. (A) Switch-like dependence on Mdm2 level arose when p53 activity increased (ΔA>0) in the presence of MdmX and low Mdm2, and when p53 activity decreased (ΔA<0) in the presence of MdmX and high Mdm2. (B, C) The effects of MdmX on p53 activity (ΔA) in a wide range of kinetic constant space were visualized. The small white circle in Figure 5-B corresponds to ΔA when k6 = 0.001 in Figure 5-A. The other small white circle in Figure 5-C corresponds to ΔA when k6 = 1 in Figure 5-A. Left-right axis: MdmX-p53 binding association constant (k25); forward-back axis: Mdm2-MdmX binding rate constant (k23); vertical axis: p53-Mdm2 binding rate constant (k11) (the dissociation rate constants were unchanged).

We hypothesized that the ability of MdmX to induce a sharp dependence of p53 activity on Mdm2 might be accounted for by the formation of MdmX∶p53 and Mdm2∶MdmX heterodimers. We tested this possibility in pre-equilibrated systems by examining the effects of heterodimer production rate constants (k11, k23, and k25) on the p53 responses to DNA damage. Transient increases of p53 activity (as detected by increased promoter occupancy, species 14) (Figure 5-A) were observed when the binding affinity (k25/k26) between p53 (species 1) and MdmX (species 5) was high or when the basal production rate (k6) of Mdm2 (species 3) was low (red area in Figure 5-B), conditions that promote a high level of p53∶MdmX (species 10) in the absence of DNA damage. In contrast, transient decreases of p53 activity (species 14) were observed when the binding affinity (k23/k24) between Mdm2 (species 3) and MdmX (species 5) was high or when the basal production rate (k6) of Mdm2 was high (blue area in Figure 5-B), conditions that promote a high level of Mdm2∶MdmX (species 9). The response patterns depicted in Figure 5B and C are consistent with the conditions in which heterodimer reservoirs served as a source of switch-like responses.

#### MdmX dampens p53 oscillation during the late response to DNA damage

**Figure 6:**
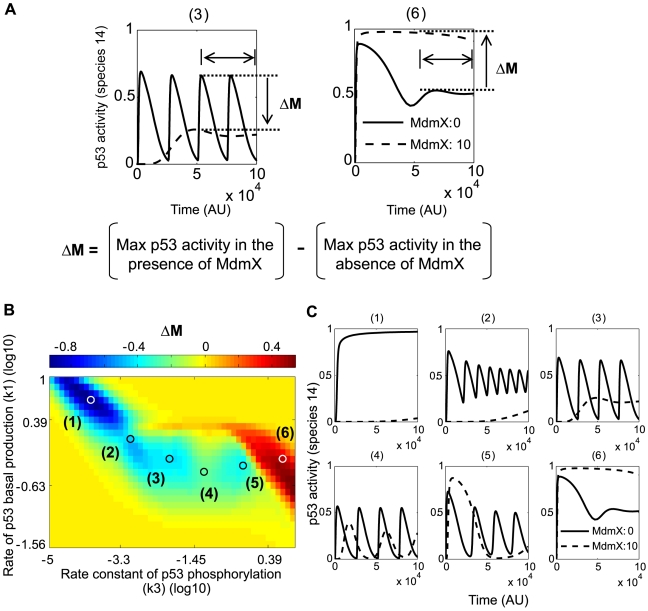
Effects of MdmX on p53 activity (species 14) during the late response to DNA damage. Simulations were based on the model in Figure 2-A. (A) Quantification of the effect of MdmX. ΔM is the difference between maximum (Max) p53 activities, in the absence and presence of MdmX, estimated during the last half of the time period. *Left*: dampening of oscillations; ΔM negative. *Right*: ΔM positive in a non-oscillating system. (B) Color-coded map of the dependence of ΔM on p53 production and phosphorylation rates. Blue area represents ΔM-negative effects of MdmX (as in examples 2 through 5 in panel C). Red area represents ΔM-positive effects of MdmX (such as example 6 in panel C). In oscillating systems, MdmX negatively regulated the oscillations, reducing the height of oscillating peaks or dampening the oscillations (panel C, (2) through (5)). In a non-oscillating system with a stable steady state, MdmX transiently reduced or elevated p53 activity (panel C, (1) and (6)), and the effects of MdmX on p53 activity were negative (C, (1)) or positive (C, (6)). The model includes p53-Mdm2 negative feedback loop (k33>0) without time delay. To measure the effects of MdmX during late DNA damage response, the system was simulated for a long period (t = 50000∼100000) under conditions that can support sustained oscillations at some combinations of p53 basal production rate and p53 phosphorylation rate constant.

Previous studies indicated that the cellular response to DNA damage involves oscillatory behaviors at the molecular level [9]. Here, the effect of MdmX on the oscillatory behavior of p53 was examined. The kinetic constants used in initial simulations for early response were varied until oscillations appeared (Table 1, column 5). In these simulations, we did not pre-equilibrate the system, because pre-equilibration did not significantly affect long-term behavior when the system was oscillating. Simulations with and without MdmX were compared in order to reveal the effect of MdmX on p53 oscillations. As output, we monitored the maximum activity of p53 achieved after a long period of time (t = 50,000∼100,000 [AU]) after DNA damage as illustrated in Figure 6A.

The results showed that, in an oscillating system, MdmX (k15 = 10) tended to dampen or reduce the amplitude of oscillations (Figure 6B and C, regimes (2) through (5)). In contrast, in non-oscillating systems with a stable steady state, MdmX could either reduce or elevate p53 activity (Figure 6B and C, regimes (1) and (6)), depending on the values of other rate constants. When lower level of MdmX (k15 = 1) was used for simulations, similar trends are observed regarding the effect of MdmX in each plot, but the degree of dampening, suppression, or positive effects is reduced in some cases (1, 4, 5, 6) (Figure S2) compared to the effects in Figure 6-C. Simulations with or without heterodimer formation showed that heterodimer formation was required to dampen oscillations (Figure S3).

**Figure 7:**
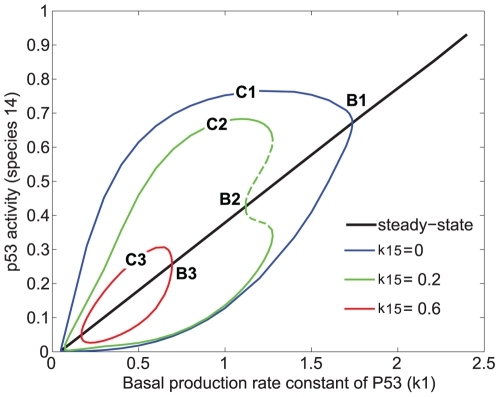
Bifurcation diagram of the effects of MdmX on p53 oscillatory behavior. Simulations were based on the model in Figure 2-A. The model includes p53-Mdm2 negative feedback loop (k33>0) without time delay and TR3 mediated positive feedback loop (k14>0). The black solid line represents steady state value of p53 activity as a function of basal production rate constant of p53. B1, B2, and B3 show bifurcation points. The steady state, surrounded by each curve (C1, C2, and C3) below the corresponding bifurcation point (B1, B2, and B3), was unstable, and the system oscillated between maximal and minimal p53 activity (species 14) indicated by each curve (C1, C2, and C3). Notably, a subcritical Hopf bifurcation was observed with k15 = 0.2, whereas supercritical Hopf bifurcations were observed when the value of k15 was 0 or 0.6. Solid curves C1, C2, C3 indicate stable limit cycles, and a dotted area in curve C2 indicates unstable limit cycles.

Next we examined the effect of p53 level on the oscillatory behavior. p53 level was varied by changing the p53 basal production rate, k1. The results are shown in a bifurcation diagram (Figure 7). The oscillation regions and amplitudes are indicated by the closed loops in the diagram. MdmX diminished both the extent of the oscillatory region and the oscillation amplitudes. At high p53 production rates (k1), the oscillations disappeared (Figure 7).

Thus, as expected, steady state of p53 activity (species 14) increases as the basal production rate constant (k1) of p53 increases (Figure 7, solid black line). In the absence of MdmX (k15 = 0), steady state of p53 activity is stable when k1 is large. However, as k1 decreases, the steady state of p53 activity loses stability at the bifurcation point B1 (Figure 7), it begins to oscillate between maximal and minimal p53 activity of the blue solid curve (Figure 7, C1). When the basal production rate constant of MdmX (k15) was increased to 0.2 or 0.6, oscillations were dampened, and the range of k1 with unstable steady states, associated with oscillatory solutions, became more limited. Notably, a subcritical Hopf bifurcation was observed with k15 = 0.2, indicating possible hysteretic behavior of the system, whereas supercritical Hopf bifurcations were observed for k15 = 0 and k15 = 0.6. MdmX also dampened oscillations of p53 activity when the p53-Mdm2 feedback loop included a time delay under oscillatory conditions (Figure S4 in Supporting Information). The role of MdmX in this oscillatory behavior has not been known, and these initial simulations with the simple model (Figure 2-A) predicted that MdmX dampened p53 oscillations.

### Surveys of kinetic parameter space

After specific behaviors of interest were identified from the initial simulations, a wider range of kinetic parameter space was surveyed with the full model (Figure 2-B) using a previously described algorithm [29] to map the variability observed in initial simulations and to test validity of the predictions made by the simple model (Figure 2-A) (see Methods). Fitness scores were designed to represent how well the model can regenerate the oscillatory patterns [9], or total protein ratio and Nutlin response data [24] (see Methods).

**Figure 8:**
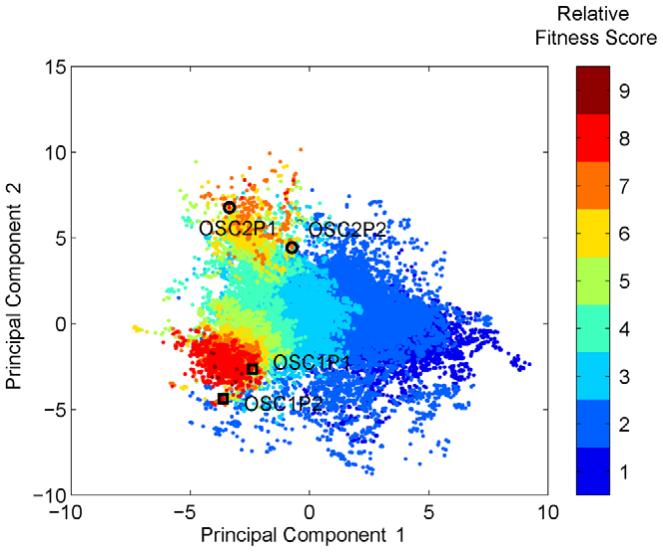
Color-coded map of kinetic parameter space based on principal components 1 and 2. Simulations were based on the model in Figure 2-B. Color in the colorbar indicates relative fitness score by binning fitness scores into nine levels. The fitness score is a measure of similarity between simulated data and the published average oscillatory pattern [9] by cross-correlation. Two oscillatory regions were identified (OSC1 and OSC2), indicated by clusters of red points. Two data points in OSC1 (OSC1P1, OSC1P2) and two in OSC2 (OSC2P1, OSC2P2) were selected for additional searches of kinetic parameter space and to fit the Nutlin response and total protein ratio.

In this search, we found regions of parameter space that exhibit oscillations and Nutlin responses that are consistent with experimental observations, and used the resulting parameter sets to simulate the effect of MdmX. These surveys were performed in two steps (see Methods). In the first step, we searched for values of 15 kinetic constants that simulate previously published average oscillatory patterns [9]. After the first step, the kinetic parameter space was visualized in two dimensions using the first and second principal components [30] (Figure 8). Two oscillatory regions (OSC1 and OSC2 in Figure 8) that gave good fits for the average oscillatory patterns were identified. In the next step, two data points were selected in each oscillatory region (OSC1P1, OSC1P2, OSC2P1, OSC2P2 in Figure 8), and these points were used as center points for the second search to fit the Nutlin response [24]. In this second search, 12 additional kinetic parameters were optimized using additional constraints.

Nutlin is a drug that blocks p53 and Mdm2 interactions, and it was shown that Nutlin induces basal transcription activity of p53 without phosphorylation [24]. When this second search was initiated from OSC2, kinetic parameter sets that fit Nutlin response were found easily. 95% of searches initiated from OSC2 gave good fits (scored≥0.25) (Table 2). However, when the search was initiated from OSC1, less than one percent of the searches gave good fits (scored≥0.25) (Table 2). The kinetic parameters that gave good fits were selected for further simulations to evaluate the role of MdmX in regulating p53 activity during early and late DNA damage responses to be described below. Simulated Nutlin responses using two example sets among the selected kinetic parameters are shown in Figure S5. Simulated oscillations and average oscillation patterns are compared for one example kinetic parameter set, and the simulated oscillations show approximately previously observed a 6 hour interval (Figure S6).

**Table 2:** Search results of kinetic parameter space and prediction of MdmX effect.

		Prediction ofMdmX effect	
Initial points	Rate of succeeded trials^a^ (succeeded trials/total trials)	Increased dependency during early response^b^	Dampening of oscillations during late response
OSC1P1	0.54% (6/1,363)	6/6 (100%)	6/6 (100%)^c^
OSC1P2	0.36% (6/1,658)	6/6 (100%)	3/6 (50%)^c^
OSC2P1	95% (169/177)	140/169 (83%)	No significant dampening^d^
OSC2P2	95% (349/368)	330/349 (95%)	No significant dampening^d^

a Searches converged to a kinetic parameter set with fitness score ≥0.25 were considered as a succeeded trial.

b The presence of MdmX (upto 100 fold k15) increased dependency by >5% increase (low Mdm2) and >5% decrease (high Mdm2) of mRNA levels (species 15).

c The presence of MdmX (upto 100 fold k15) decreased maximum value (peak of oscillations) by >30%.

d The decrease of maximum value (peak of oscillations) was below 5% in the presenece of MdmX (upto 100 fold k15).

Sensitivity analyses were performed for two selected sets of kinetic parameters that were used for further simulations. The normalized local sensitivity of mRNA (species 15 in Figure 2-B) with respect to various parameters was calibrated during an early DNA damage response (Figure S7), and the time integral of the local sensitivity values were used to rank order the parameters based on their mRNA sensitivity (species 15 in Figure 2-B) (Table S3-A,B). In general, the two kinetic parameter sets showed similar levels of sensitivities. In both sets, the most sensitive kinetic parameters were parameters involved in p53 oligomerization and were involved in the production and degradation of p53 and Mdm2. In the kinetic parameter set derived from OSC1, parameters relevant to the production and degradation of p53 and Mdm2 were most sensitive; in the kinetic parameter set derived from OSC2, parameters relevant to p53 oligomerization were most sensitive.

### Simulations with a full model and fitted parameter sets

#### MdmX can induce switch-like behaviors

**Figure 9:**
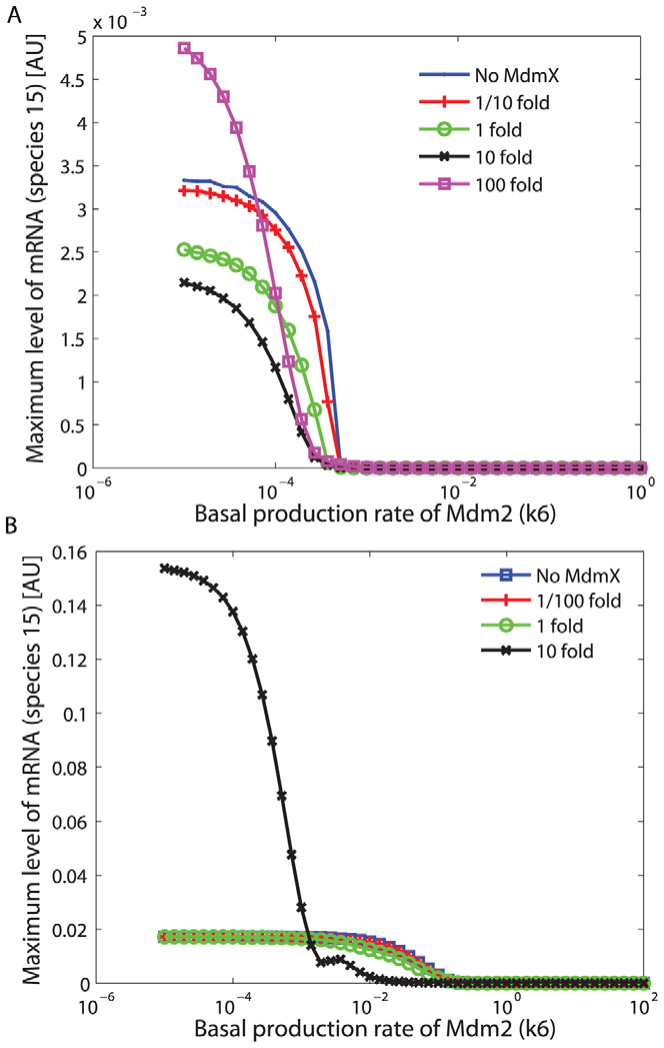
Early response to DNA damage simulated using the kinetic parameter sets derived from OSC1P1 and OSC2P1. Simulations were based on the model in Figure 2-B with kinetic parameter sets derived from (A) OSC1P1 and (B) OSC2P1. Maximum level of mRNA (species 15) was quantified during early DNA damage response (0-3 h) as a function of increasing basal production rate constant of Mdm2 (k6) in the presence of different levels of MdmX. The green line with open circles (also indicated as 1 fold) corresponds to a predicted basal production rate of MdmX from the survey of kinetic parameter space. The basal production rate of MdmX was varied from 0 to 100 fold of its predicted value.

Simulations for early DNA damage response were performed as described in method. The maximum level of p53-induced mRNA (species 15) during the first 180 min post DNA damage was considered as p53 activity and measured as output while basal production rate of Mdm2 (k6) was varied. Examples of the transient dynamic behavior observed when the production rates of Mdm2 and MdmX were varied are shown in Figure S8-C (Supporting Information). As observed in Figure 3, Mdm2 altered p53 activity more severely in the presence of MdmX (Figure S8-C). Simulations using two parameter sets, derived from OSC1P1 and OSC2P1, are shown in Figure 9. As shown in Figure 4 and Figure 5-A with the simple model, MdmX increased dependency of p53 on Mdm2; in the presence of 100 fold MdmX, the p53 activity was either elevated or suppressed depending on the level of Mdm2 when compared to the response without MdmX (Figure 9). For some Mdm2 basal production rates below 10^−4^, the p53 activity could be increased over 30% in the presence of MdmX (Figure 9-A); for some Mdm2 basal production rates above 10^−4^, the p53 activity could be decreased over 50% (Figure 9-A). Such Mdm2 dependent MdmX effects, which can induce switch-like behaviors, were observed in most (>83%) of the used kinetic parameter sets (Table 2) at some level of MdmX (Figure 9). As shown in Figure 5, association rate constants (k23 and k25) of Mdm2∶MdmX and p53∶MdmX can affect the switch-like behaviors (Figure S8).

#### Variability of MdmX-induced dampening

**Figure 10:**
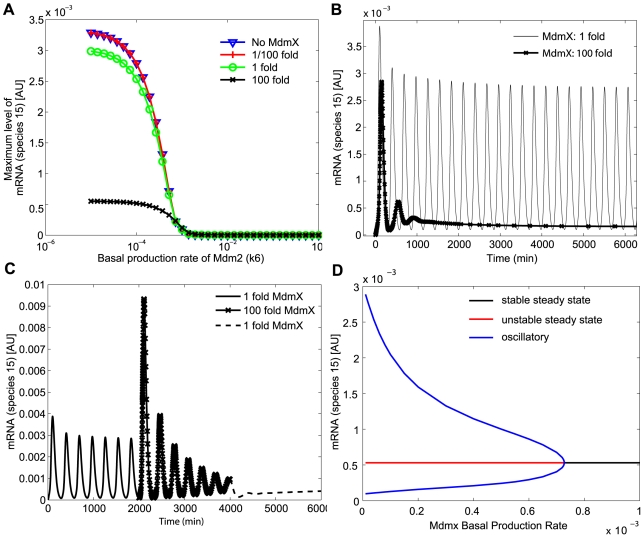
Prediction of late response to DNA damage. Simulations were based on the model in Figure 2-B. One of the kinetic parameter sets derived from OSC1P1 was used for simulation. (A) Maximum level of mRNA was quantified during late DNA damage response (t = 10,000∼11,000 min) as a function of increasing basal production rate of Mdm2 (k6) in the presence of different levels of MdmX. The green line with open circles corresponds to predicted basal production rate of MdmX from the survey of kinetic parameter space. The basal production rate of MdmX was varied from 0 to 100 fold of its predicted value. (B) Dampening of oscillations by increased basal production rate of MdmX is shown as a function of time. (C) Persistent dampening of p53 oscillation by MdmX. First, the system was simulated with a low level of MdmX basal production rate for 2000 min; the system exhibited sustained oscillations. The basal production rate was then increased 100-fold, and simulation was continued for additional 2000 min (t = 2000 to 4000 min); the oscillations were damped, and the system approached steady state conditions. Then the basal production rate was decreased to its original value, and the system was simulated for another 2000 min (t = 4000 to 6000 min); oscillatory behavior was suppressed over this extended period of time. The bifurcation diagram (D) of p53 activity as a function of MdmX basal production rate suggests that the transient change in the MdmX basal production rate takes the system from conditions near a stable oscillatory state to a stable steady state. Subsequent reduction of basal production rate of MdmX to its original value eventually recovers oscillatory behavior.

Late DNA damage response (t = 10,000 to11,000 min after DNA damage) with a full model (Figure 2-B) was examined using the kinetic parameter sets found from the search. When kinetic parameters chosen from OSC1P1 or OSC1P2 (Table 2) were used, simulations with the full model predicted that p53 (species 15) oscillations were dampened by a 100-fold increase of the basal production rate constant of MdmX (Figure 10) as observed in initial simulations (Figure 6, 7) with simple model (Figure 2-A). In contrast, when kinetic parameters derived from OSC2P1 and OSC2P2 (Table 2) were used, dampening of oscillations was not observed at any concentration of MdmX (see Figure S9). However, when oscillations were suppressed by 100-fold increase of MdmX basal production rate, a subsequent reduction of MdmX basal production rate was followed by a long period of continued suppression before oscillations were recovered, as shown in Figure 10-C, D. Thus transient high expression of MdmX may confer extended periods of suppressed oscillatory behavior of the system.

## Discussion

### Extraction of a core sub-network for simulation

In order to investigate the role of MdmX in the p53-dependent response to DNA damage, we prepared a heuristic MIM of the pathways leading from DNA damage to p53 activity (Figure 1), from which we selected interactions for an explicit MIM (Figure 2) that defines the network to be simulated.

The known pathways of p53 regulation are highlighted in four panels in Figure 1. The pathway steps highlighted in Figure 1 were all included in the simulation model displayed in Figure 2, except for the following:

1. Enhancement by p53 multimerization of the binding between p53 and Mdm2 [A13].
2. Phosphorylation by Casein Kinase 1 alpha and its effect in binding between p53 and MdmX [A19, A20].
3. Autoubiquitination of Mdm2 (it was recently reported that Mdm2 does not regulate its own stability [31], moreover, the possible involvement of the third molecule inducing Mdm2 ubiquitination and degradation is consistent with our model.)
4. The effects of Hausp were simulated by more rapid degradation of unphosphorylated than phosphorylated Mdm2 or MdmX either by basal degradation or Mdm2 dependent degradation except in parameter sets for Figure 6, 7 (Table 1) [A9, A10, A12, A23, A29, A30, A33, A34, A39, A40].
5. The interactions of potential p53∶Mdm2∶MdmX ternary complexes were omitted from the model.

A recent mouse study suggested that p53 phosphorylation may be less important for stabilization and activation of p53 than previously thought [20]. This however remains consistent with our model, in which stress-dependent modification (phosphorylation and/or ubiquitylation) of p53, Mdm2, and MdmX operate coherently to Sstabilize and activate p53.

For simulations, two versions of the network model were used: a simple network model (Figure 2-A) and a full network model (Figure 2-B). These two versions differ with respect to the basal transcription activity of p53 (k34, k35, k36), p53 induced Mdm2 production (k33, k37, k38) and mRNA (species 15): the simple network model does not include the p53 basal production rate and the mRNA species (in that case, species 14 directly generates Mdm2, species 3). Initial simulations were performed with the simple network model (Figure 2-A), because it allowed us to interpret simulation results more easily. The biologically interesting network behaviors, found from the initial simulations, were further surveyed in a wider range of kinetic parameter space using a full network model including basal p53 activity and mRNA (Figure 2-B).

### MdmX dependent switch-like behaviors

Reliable values for rate constants that would apply to the peculiar and diverse conditions existing in cells are elusive, because local molecular crowding in different regions of the cell can substantially affect the thermodynamic parameters [32], and conditions are likely to vary with time and place in the cell. That difficulty exists for the cell as well as for the computer modeler, and may be ameliorated for both if the networks operated in a somewhat digital mode, that is to say with switch-like behavior. Then connections in the network would tend to be either on or off, and quantitative degree of function would be less of an issue. In fact, switch-like behavior has been observed in biological systems [33]–[35]. The problem could also be mitigated if the range of parameter values existing in the cell were in a region of parameter space where function is not much changed over local regions in the parameter space. Network function would then be robust in being relatively invariant over a region of parameter space. Moreover, functional invariance over a region of parameter space would be a condition relatively easy for evolution to access, and therefore would be more likely to exist in modern cells. Based on these considerations, we focused our attention on switch-like behavior and exploration of parameter space. We have also centered some of our studies at plausible sets of parameter values, derived from published experimental data.

**Figure 11:**
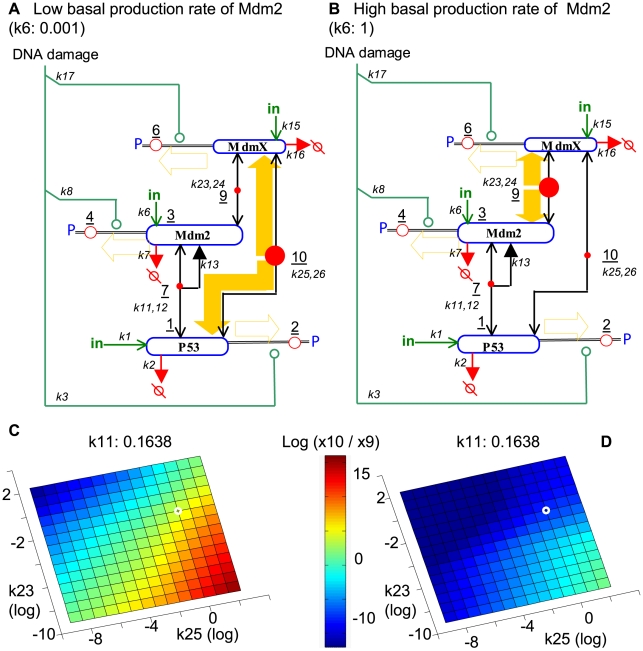
p53:MdmX and Mdm2:MdmX as reservoirs of p53 and Mdm2 during early DNA damage response. Simulations were based on the model in Figure 2-A. The system was equilibrated prior to DNA damage. (A, B) The relative levels of p53:MdmX and Mdm2:MdmX reservoirs at steady state are represented qualitatively by the sizes of the solid red circles in panel A and B. The open red circles represent phosphorylated species, which are induced by DNA damage and are absent prior to DNA damage. (C, D) The relative levels of two reservoirs, p53:MdmX and Mdm2:MdmX, are shown at t = 55 after DNA damage. Reservoir size was compared by log ratio ( log(x10/x9) ) and visualized using a color-coded image map/heat map. Figure 11-A corresponds to the small white open circle in Figure 11-C; Figure 11-B corresponds to the small white open circle in Figure 11-D. (A) When the basal production rate of Mdm2 is low, the level of p53:MdmX reservoir can accumulate during pre-equilibration because the rate of Mdm2 dependent degradation of p53 is low and relatively more p53 can exist. (B) In contrast, when the basal production rate of Mdm2 is high, the level of Mdm2:MdmX reservoir can accumulate during pre-equilibration because the rate of Mdm2 dependent degradation of p53 is high and relatively low p53 exist. Left-right axis: MdmX-p53 binding association constant (k25); forward-back axis: Mdm2-MdmX binding rate constant (k23) (the dissociation rate constants were unchanged).

The switch-like behavior induced by MdmX in our simulation was dependent upon the heterodimers p53∶MdmX and Mdm2∶MdmX. When DNA damage-induced phosphorylation perturbs the system by depleting free p53, Mdm2, and/or MdmX, the heterodimers dissociate to compensate those depletions. The dissociation of the two MdmX heterodimers, p53∶MdmX and Mdm2∶MdmX, have opposite effects on p53 activity; therefore, the net effect on p53 activity depends on the relative level and contribution of those heterodimers. In initial simulations, switch-like behavior occurred when p53∶MdmX (species 10) became a major reservoir during the pre-equilibration with low Mdm2 or when Mdm2∶MdmX (species 9) became a major reservoir during the pre-equilibration with high Mdm2 (Figure 11-C, D). Figure 11 illustrates the idea using an initial model. Most simulations (over 80%) using the fitted parameter sets with a full model also generated Mdm2 dependent MdmX effects suggestive of reservoir-based amplification during an early response.

Unlike some previously reported switch-like behaviors arising from steady states [34],[36],[37], memory-less switch-like behavior was observed during transient response in our models. This kind of switch may usefully generate rapid transient responses to perturbations when the system has not had time to reach steady state. The switch-like dependence of p53 activity on Mdm2 may produce different degrees of susceptibility to DNA damage in various cell states and cell types. The question of whether such reservoir-based switch-like behavior is employed during response to DNA damage could be addressed by measuring p53 activity at different levels of Mdm2 and MdmX early during the response.

### MdmX can dampen oscillations

Previous studies showed sustained oscillations of p53 during the long-term response to DNA damage [9], [38]–[40]. In the mathematical models described here, either time delay or TR3-mediated positive feedback was required to display sustained oscillations, consistent with a previous study [10]. TR3 promotes Mdm2 self-ubiquitination in a p53-dependent manner [41], and the relation may form a positive feedback loop. The feedback loop via TR3 was implied by k14 in Figure 2. Our initial study with a set of kinetic parameters that generated oscillations showed that increased level of MdmX can dampen oscillation via non-enzymatic interactions. The reason may be that the concentrations of the unbound p53 and Mdm2 were buffered by the p53∶MdmX and Mdm2∶MdmX heterodimer reservoirs. When some kinetic parameters were varied, sometimes p53 activity converged to a stable steady state without oscillations; in this system with a stable steady state, MdmX transiently affected p53 activity in either a negative or positive manner, but eventually p53 activity converged to the same stable steady state. The simulation with full model also showed damped oscillations with the increased MdmX with some sets of parameters. Interestingly, with some kinetic parameter sets, transient increases of MdmX levels dampened oscillations over an extended time period, with sustained quiescent intervals following the reduction of MdmX to prior levels. The bifurcation diagram of p53 activity as a function of MdmX basal production rate suggests that the transient change in the MdmX basal production rate takes the system from conditions near a stable oscillatory state to a stable steady state. The suppression of oscillations following the return to the original production rate is likely a ‘memory effect’ in which the system tracks back along the steady state solution branch, ending in the vicinity of an unstable equilibrium point where the oscillatory instability is slow to build up.

### Survey of parameter space

Kinetic parameter space was explored in two stages. (1) During the first stage, two oscillatory regions, OSC1 and OSC2, were identified. The characteristic difference between the OSC1 and OSC2 clusters was the phosphorylation rate constant (k3 in Figure 2). Narrow distributions of k2, k10, and k37 were common in both clusters, which may mean that oscillatory behavior is sensitive to the degradation rate of p53, Mdm2, and mRNA (see Figure S10). (2) In the second step, two parameter sets from each region were selected as center points to search neighbor parameter space to fit experimental data with a new fitness function. The searches showed marked difference depending on the initial center point in their success rates (a ratio to converge to the second criteria, Nutlin response and total protein ratios in MCF7 cells), and OSC2P1 and OSC2P2 were better center points than OSC1P1 and OSC1P2. Notable, only when the model was simulated with the parameter sets derived from OSC1 were oscillations dampened by MdmX. Thus there may be two separate clusters of kinetic parameters corresponding to p53 oscillations during the late response to DNA damage: kinetic parameter sets derived from OSC1 are MdmX-sensitive, while kinetic parameter sets derived from OSC2 are MdmX-insensitive. Since negative regulation of p53 oscillations by MdmX correlates with parameter sets derived from OSC1, it is tempting to conclude that models defined by these kinetic constants best describe the regulatory behavior of the p53-Mdm2–MdmX network. However, it is not obvious how nature could have evolved such a finely tuned set of parameters considering the small success rate (<0.5%) in fitting Nutlin and total protein ratio data.

The two groups of parameter sets derived from OSC1 or OSC2 underlie MdmX-sensitive and MdmX-insensitive models. It is likely that physiological condition will be explained better by one of those kinetic parameter sets. Experiments to test whether the over-expressed MdmX can in fact dampen DNA damage induced p53 oscillations will allow us to discriminate between the two models.

Our theoretical study showed, with little prior knowledge, potentially interesting roles of MdmX in p53 regulation. Many of the kinetic constants used in the study were not biologically constrained, and some of the assumptions made in the models need to be biologically validated. The lack of prior knowledge demands rigorous experimental validation of the simulated results; however, it also allows the unbiased investigation of possible functions that are not intuitively obvious. The MdmX roles predicted in this study are not simply linear increases or decreases of p53 activity dependent upon the level of MdmX; the study suggests that manipulation of MdmX to alter p53 activity requires careful investigation of the dynamics of three proteins: MdmX, Mdm2, and p53. An experimental system with inducible MdmX and Mdm2 and a reporter protein to monitor p53 activity should allow us to test whether the switch-like behavior (increased dependency on Mdm2 by MdmX) or dampening of oscillations are observed. To observe the switch-like behavior, transient p53 activity has to be monitored, and it may require monitoring the p53 activity relative early (10, 30, 60, 120, 180 min) after DNA damage. To observe dampening of oscillations by MdmX, a long period of observation (>12 hr) will be necessary since the known peak-to-peak interval is approximately 6 hours. As shown in one example plot in the result section with the full model, the degree of level change in the switch-like effects by the increased MdmX was approximately 30%∼50% increase (low Mdm2) or decrease (high Mdm2) of p53 activity, and that level of change may require high sensitivity to measure p53 activity in experiments.

We used highly simplified p53 models in this study because we can interpret the simulation results more easily. However, p53 is involved in many other regulations and feedback loops, and it is not obvious whether the conclusions will still hold in a larger p53 system. Although experimental verification will partially address the question, we could also proceed theoretically by introducing additional components successively to the current model and by surveying parameter space with an updated fitness function. The extension of the current model might include Wip1, which is known to trigger recurrent initiation of ATM activity in some cells [13], detailed phosphorylation and dephosphorylation of molecules, degradation of p53 by ubiquitination steps, and additional interacting partners with Mdm2 and MdmX. Those interactions may introduce additional switch-like steps [42],[43], and it may be interesting also to investigate how dynamic behaviors change when these multiple switches operate together in the same system.

One goal of this study was to understand how non-enzymatic interaction of MdmX with p53 and Mdm2 can regulate p53 during the response to DNA damage. The mechanism of p53 regulation by MdmX is poorly understood, at least in part because the role of Mdm2∶MdmX heterodimer in p53 ubiquitination and degradation is not clearly understood. It was previously speculated that p53∶MdmX heterodimer may serve as a reservoir to maintain a pool of p53 [44]. The findings reported here suggest how such biological reservoirs, which lack any enzymatic activity, can account for previous studies that proposed negative regulation [21]–[23] and various effects [16]–[18] observed on p53 activity by MdmX. Furthermore, the results suggest that heterodimer reservoirs can differentially alter dynamic behaviors during short- and long-term responses to system perturbations. Lastly, this study uncovers a new potential mechanism for inducing a switch-like behavior of p53, which operates transiently in response to cellular events such as DNA damage and depends on heterodimer reservoirs. These results can guide future experiments to elucidate mechanisms by which Mdm2 and MdmX may regulate p53 responses to DNA damage.

## Methods

### Model construction

First, a heuristic molecular interaction map (MIM) (Figure 1) was constructed by using a previously described notation [25]–[27]. Next, we extracted from the heuristic MIM a sub-network in the form of an explicit MIM that defines a computer simulatable model (except for parameter values and initial conditions) (Figure 2). In an explicit map of the model, all interactions and contingencies are displayed as molecular association/dissociation or as stoichiometric conversions and can be represented as reactions. A list of reactions obtained from the explicit map (Figure 2) was used to write ordinary differential equations (ODEs) (Table S4), which mathematically describe the p53-Mdm2-MdmX network model, based on mass action law. The model was simulated by numerically solving the ODEs (using ODE15S or DDE23 functions) in Matlab.

### Kinetic constants for initial simulations

The kinetic constants used for initial simulations were generated based on collected kinetic constants from previously published models [8],[10],[39] (see Table 1 and Table S5). For kinetic constants whose values were not available from previous models, values were chosen randomly based on the assumptions in Table S6. Although reported qualitative behaviors of molecular interactions support some of the assumptions in Table S6, many of them were assumed for simplicity. Some of the kinetic constants that were available from previous models differed over a wide range and did not represent equivalent processes. We therefore chose values randomly within or near the range of collected values to generate an initial set of kinetic constants. We simulated the network by varying one or more kinetic constants from the initial set to understand the effects on network dynamics. The assumptions in Table S6 were relaxed when the kinetic constants were varied, and the used kinetic parameter set for initial simulations are presented in Table 1.

### Input and output for initial simulations with the simple model (Figure 2-A)

As input, rate constants of phosphorylation (k3 = k8 = k17) and basal production rate of MdmX (k15 in Figure 2) and Mdm2 (k6) were varied. As output, p53 activity was quantified as p53-dependent promoters occupied by p53 tetramers (species 14 in Figure 2). In these initial simulations with the simple model (Figure 2-A), p53 tetramers were assumed to be the only form of p53 that can bind promoters (tetramer-occupied promoters are represented by species 14 in Figure 2). In the model used here, MdmX modified transient dynamics of p53 activity without altering the p53 steady state activity, as can be deduced from the equations and verified in the numerical solutions. Output was therefore collected at a fixed time point regardless of whether or not the system had reached a steady state. Simulation results with the simple model were presented as [AU] because the kinetic parameters were not fit to any experimental data.

### Simulation of an early and late DNA damage response

We considered that DNA response may display bi-phasic responses: early and late responses. During initial simulations with the simple model (Figure 2-A), early response was measured at t = 55 [AU] after DNA damage. The time t = 55 [AU] was chosen because the system did not reach steady state and various types of system responses were observed depending on the choice of kinetic parameters at this time (Figure 3, 4, 5, 11). These initial simulations were performed without p53-Mdm2 transcription feedback to interpret simulation results more easily. For late response, the system was simulated for a long period of time until multiple numbers of sustained oscillatory peaks were observed (Figure 6, 7, 8, 9). In subsequent simulations with the full model, early response was measured by the maximum level of mRNA achieved during 0∼180 min after DNA damage. For late response, the system was again simulated for a long period of time until multiple numbers of sustained oscillatory peaks were observed.

To set realistic initial condition for early DNA damage response, cells without DNA damage were simulated by pre-equilibrating the model in the absence of DNA damage induced kinase activities (k3 = k8 = k17 = 0). For late DNA damage response, simulations were performed without pre-equilibration. DNA damage was simulated by setting all the rate constant of phosphorylation as a same positive value (k3 = k8 = k17>0) unless otherwise indicated. The rate constant of phosphorylation was kept constant during the simulation of DNA damage response because quite rapid saturation of ATM activity followed by a constant level of ATM activity up to 24 hr were observed in a previous study [45].

### Computation of Figure 7 and Figure 10-D (bifurcation diagram)

The bifurcation diagram shown in Figure 7 was generated for the parameter set given in column 5 of Table 1, with k3 = 0.0086851. Steady state values of the species in the system can all be expressed in terms of the steady state value of p53, which satisfies a single nonlinear equation. The steady state values of all of the species that do not involve MdmX were found to be independent of the levels of MdmX. The stable steady state values shown in the diagram were readily computed by integrating the ODEs for long times until transient effects decayed. The branch of both stable and unstable steady states was then computed using a quasi-Newton method with continuation along the branch from stable values. The linear stability of the steady state solution was determined by computing the eigenvalues of the Jacobian matrix at each point. The stable oscillatory solution branches could also be computed by integration of the ODEs for long times. In addition, both stable and unstable (subcritically bifurcating solutions) oscillatory solutions were computed by a shooting method that enforces periodic boundary conditions over a single period of oscillation. Continuation was used when necessary to follow solution branches from stable to unstable regions of parameter space.

### Survey of kinetic constants

To search parameter space, we used a previously described algorithm [29]. Briefly, the algorithm first randomly selects an initial point as a center point in a k dimensional space (where k is the number of rate constants) and randomly searches for neighbor points within a radius R. Once a point with a higher fitness score is found, the center point is updated. The method is iteratively repeated with larger values of R until the searching criteria (high fitness score) is satisfied.

**Figure 12:**
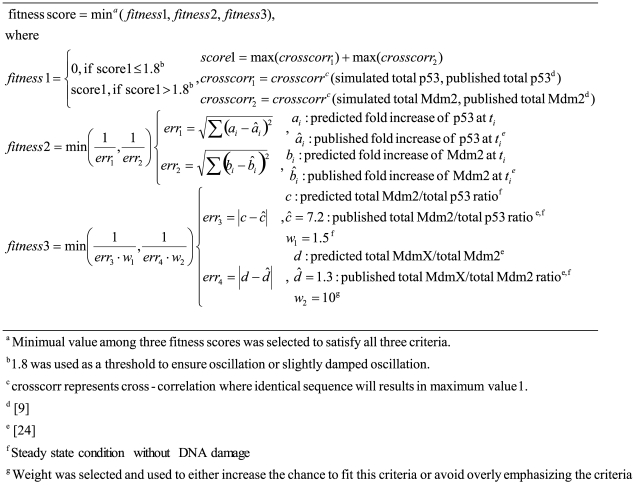
Definition of a fitness function used in the second step during the parameter space search.

The searches were done in a two step procedure. First, a subnetwork including a subset of interactions and kinetic constants was selected, based on their previously predicted roles in oscillatory behaviors [38],[39]. This subnetwork included 15 unknown kinetic constants (k2, k3, k10, k11, k12, k13, k27, k28, k29, k30, k31, k32, k33, k37, k38), and we searched this 15 dimensional parameter space for oscillatory behaviors. To limit the extent of parameter space to search, we assumed k7 = k5 = k2, k14 = k13*0.2, k8 = k3, k9 = k4 = k3*0.02. During the search, fitness scores were calculated based on cross-correlation [30] between simulated time series data and a published average oscillation pattern [9]. Among the high scored sets of parameters, we selected sets of kinetic constants that generated oscillations with certain maximum values of p53 activity (species 14>0.0001 [AU]). The selected kinetic sets were used as initial center points for the next searches to fit additional experimental data. In this second search, we searched 12 dimensional space (k2, k3, k6, k7, k15, k16, k23, k25, k34, k36, k38). To limit parameter space, we assumed k10 = k17 = k3, k4 = k9 = k18 = k3*0.02. The remaining kinetic constants, determined from the first search, were retained during the second parameter search. In the second search, fitness scores were calculated based on three types of published data: average oscillatory patterns [9], Nutlin response [24], and Mdm2/p53 and MdmX/Mdm2 total protein ratios [24] (Figure 12).

### Sensitivity analysis

The normalized local sensitivity of mRNA (species 15 in Figure 2-B) with respect to various kinetic parameters 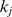 was defined by the following equation
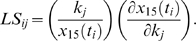



The local sensitivity calculation was performed using the Simbiology toolbox in Matlab. The time integral (*LS_j_*) of the normalized local sensitivity was obtained by trapezoidal numerical integration [46] and used to rank the parameters based on their sensitivity value,
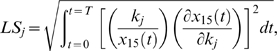
where T = 180 was chosen as the length of the interval of integration.

### Simulation of DNA damage response with the full model (Figure 2-B) using surveyed kinetic parameters

DNA damage was simulated similarly as in initial simulations. As output, species 15 (mRNA) was measured because the full model include basal p53 activity (transcription activity by p53 monomer and promoter complex). Early DNA damage response was quantified by the maximum p53 activity (species 15) achieved during 0∼180 min after DNA damage; this period corresponds to the first rise in p53 activity after DNA damage [47]. Late DNA damage response was quantified by the maximum p53 activity achieved during 10020 min∼10980 min (time window arbitrarily chosen after long period of simulation) after DNA damage. Such long term simulations were performed to allow the initial trajectory to converge to a limit cycle from an initial condition. Maximum p53 activity was taken to be the maximum level of mRNA (species 15 in Figure 2) achieved during the indicated time frame. In the initial simulations for early DNA damage response, we measured p53 activity at one time point. In the subsequent simulations (Figure 3, 4, 5), however, we selected the maximum values of p53 activity during a time window 0<t<180 min to avoid any sensitivity to a single measurement time (Figure 9). To measure the effects of MdmX, we compared the maximum p53 activities achieved in simulations with and without MdmX. When MdmX was included in the model, various levels (1/100, 1/10, 1, 10, 100 fold) of the basal production rate constant (k15) of MdmX were used.

### Simulated Nutlin response without DNA damage

The network was pre-equilibrated without Nutlin (k11>0) and without DNA damage (k3 = k8 = k17 = 0). For the simulation of Nutlin treatment, we simulated a network with k11 = 0, because Nutlin is known to affect Mdm2-p53 interaction only [48]. The fold increase in total p53 and Mdm2 was measured as a function of time. To fit Nutlin response data, it was assumed that unphoshorylated p53 could display basal Mdm2 induction activity [24].

### Matlab scripts

Some of example matlab scripts used for simulations were provided as supplemental information (Text S1, S2, S3, S4, S5, S6, S7).

## Supporting Information

Figure S1 An informal diagram of Figure 2B based on the diagram convention used by Wahl (Cell Death and Differention 13:973).
(1.11 MB EPS)

Figure S2 Regenerated example plots in Figure 6 with MdmX = 1. Simulations were based on the model in Figure 2-A. The effect of MdmX on the oscillatory behavior of p53 was examined. The kinetic constants used are in Table 1, and the used initial condition is in Table S2. Simulations with and without MdmX were compared in order to reveal the effect of MdmX on p53 oscillations. Each plot corresponds to the plot in Figure 6-C. In general, similar trends are observed regarding the effects of MdmX in each plot, but the degree of dampening, suppression, or positive effects is reduced in some cases (1, 4, 5, 6) compared to the effects in Figure 6-C.
(0.08 MB TIF)

Figure S3 Effects of p53∶MdmX and Mdm2∶MdmX heterodimer reservoirs on p53 oscillatory behavior. The model is based on Figure 2A including the p53-Mdm2 negative feedback loop (k33>0) without time delay and also includes the TR3 mediated positive feedback loop (k14>0). The used kinetic parameter set is listed in Table 1. Simulations were performed with various levels of the basal production rate constant (k15) of MdmX with (k23 = k25 = 0.12) and without (k23 = k25 = 0) heterodimer formation. Maximal (Max) p53 activity was evaluated during the last half (t = 250000 to t = 500000 [AU]) of the simulated period, while the basal production rate constant (k15) of MdmX was varied. (A) p53 activity was estimated with (blue line) and without (red line) formation of p53∶MdmX and Mdm2∶MdmX heterodimers. The p53 activity curve obtained from a model including formation of heterodimers has three characteristic areas indicated as B (no dampening of oscillations), C (reduced oscillatory peaks), and D (dampened oscillations). Time simulation data corresponding to B, C, and D area of curves in (A) are shown in panel (B), (C), and (D) respectively. The dampening effect of MdmX on oscillations was not observed when p53∶MdmX and Mdm2∶MdmX heterodimers were excluded from the model (k23 = k25 = 0). For kinetic constants used, see Table 1.
(0.64 MB TIF)

Figure S4 MdmX-induced reduction of the amplitude of oscillating p53. This simulation included the p53-Mdm2 feedback loop (k33>0) with a time delay and without the TR3-mediated positive feedback loop (k14 = 0), and the model is based on Figure 2-A. The thin line represents simulation without MdmX (k15 = 0). The dark line represents simulations with MdmX production rate at k15 = 0.1. For kinetic constants used for simulation, see Table 1.
(0.28 MB TIF)

Figure S5 Comparison of experimental and simulated Nutlin responses. Simulations were performed based on the model in Figure 2-B. Kinetic parameter sets derived from OSC1P1 (Figure 8) (A) and OSC2P1 (Figure 8) (B) were used for simulation (see Table 1). Fold-increases in total levels of p53, Mdm2, and MdmX are shown as a function of time after Nutlin treatment. The experimental values were taken from Figure 5 in Wang et al. (PNAS, 2007).
(1.35 MB EPS)

Figure S6 Simulations were performed based on the model in Figure 2B. Simulation results with one set of parameters used for Figure 10 (Table 1) were plot against the averaged oscillatory pattern. To fit to the experimentally observed oscillation period, first, we manually extracted data points from the average oscillation pattern (Fig. S6 in Geva-Zatorski et al. 2006 MSB). Next, we searched for a parameter set which produces approximately similar oscillation interval with the averaged oscillation pattern, and the fit was evaluated by cross-correlation. With the parameter set used in Figure 10, the first peak of total p53 in simulated data appeared and peaked earlier than the averaged p53 oscillation pattern because cross-correlation does not capture whether the signal begins at specific time point or not. Given the observed variability in oscillations (Geva-Zatorski et al. 2006 MSB), we did not intend to fit the exact time point of each peak. The following figures show the overlay of averaged oscillation pattern of each protein and simulated data. For better comparison, the simulated data were plotted by shifting 50 min. For the simulations in the following two figures, same initial condition in Table S2 was used.
(0.14 MB TIF)

Figure S7 The normalized local sensitivity (*LS_ij_*) of mRNA (species 15 in Figure 2B) with respect to various parameters. Among 38 kinetic parameters, the 12 most sensitive parameters based on the time integral (see Materials and Methods) were plotted for (A) parameters derived from OSC1 (column 8, Table 1) and (B) parameters derived from OSC2 (column 9, Table 1).
(0.21 MB TIF)

Figure S8 Region of switch-like behaviors as a function of association rate constants of p53∶MdmX and Mdm2∶MdmX. Simulations were performed based on the model in Figure 2-B with pre-equilibration. Log ratio of two p53 activities (with and without MdmX) in Mdm2 = 0.00005 (A) and in Mdm2 = 0.0002 (B) were visualized. Two association rate constants, k23 and k25, were varied from the parameter set used for Figure 9-A (Table 1). The white circle corresponds to the simulation with a parameter set for Figure 9-A. The figure visualizes the log ratio of the p53 activity without MdmX and p53 activity with 100 fold MdmX. The figure shows that when the binding affinity (k25) between p53 and MdmX becomes less tight, the presence of MdmX has negative effect on the p53 activity in a wide range of k23. Also, the figure shows that the switch-like behavior is observed in a wide range of k23 (along the vertical line of white circle) where the yellow (left panel) and blue (right panel) areas overlap. The time series data that correspond to the kinetic parameter sets of white circle (A, B) are shown in panel (C). The time plot shows that the dependency of p53 on Mdm2 increased in the presence of MdmX.
(0.26 MB TIF)

Figure S9 Prediction of late response to DNA damage. Simulations were performed based on the model in Figure 2-B. One of kinetic parameter sets derived from OSC2P1 was used for simulation (Table 1). Maximum level of mRNA was quantified during late DNA damage response (t = 10,000∼11,000 min) as a function of increasing basal production rate of Mdm2 (k6) in the presence of different levels of MdmX. The green line with open circles corresponds to a predicted basal production rate constant of MdmX from the survey of kinetic parameter space. The basal production rate constant of MdmX was varied from 0 to 100 fold of its predicted value.
(0.81 MB EPS)

Figure S10 Distribution of each kinetic parameter in cluster OSC1 (A) and OSC2 (B) in Figure 8 (column order: k2, k11, k12, k13, k3, k29, k30, k31, k32, k27, k28, k38, k10, k33, k37).
(0.18 MB TIF)

Table S1 Annotations of Mdm2-p53-MdmX MIM.
(0.13 MB DOC)

Table S2 Initial condition.
(0.04 MB DOC)

Table S3 Rank ordered time integral (LSj) of the normalized local sensitivity of the kinetic parameter sets from (A) Table 1 column 8 and (B)Table 1 column 9.
(0.11 MB DOC)

Table S4 Ordinary differential equations for figure 2-B (symbols are in Table 1).
(0.03 MB DOC)

Table S5 Kinetic constants used in previous mathematical models.
(0.04 MB DOC)

Table S6 Assumptions used to generate an initial kinetic parameter set (column 3 in Table 1).
(0.03 MB DOC)

Text S1 Matlab script: Parameter definition to be used to generate figure 4.
(1 KB TXT)

Text S2 Matlab script: Function definition for a simple model.
(2 KB TXT)

Text S3 Matlab script: Simulate and plot figure 4.
(0.01 MB TXT)

Text S4 Matlab script: Simulate and plot Figure 6-B and C.
(0.01 MB TXT)

Text S5 Matlab script: Function definition for the full model.
(3 KB TXT)

Text S6 Matlab script: Function required to generate figure 9.
(2 KB TXT)

Text S7 Matlab script: Simulate and plot figure 9.
(3 KB TXT)

Text S8 SBML format model. Define the full model (Figure 2-B) in SBML format with a kinetic parameter set in column 9, Table 1. The sbml model definition was generated from the Simbiology toolbox in Matlab.
(0.04 MB SBML)
