# Supplementary figures and images for: Predicted Functions of MdmX in Fine-Tuning the Response of p53 to DNA Damage
Source: PLoS Comput Biol. 2010 Feb 5;6(2):e1000665. doi: 10.1371/journal.pcbi.1000665 (PMC2824598; doi:10.1371/journal.pcbi.1000665)

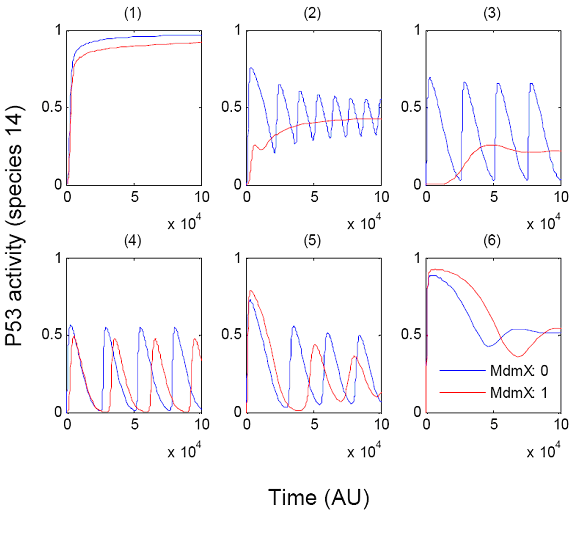

Supplement: Figure S2 — Regenerated example plots in Figure 6 with MdmX = 1. Simulations were based on the model in Figure 2-A. The effect of MdmX on the oscillatory behavior of p53 was examined. The kinetic constants used are in Table 1, and the used initial condition is in Table S2. Simulations with and without MdmX were compared in order to reveal the effect of MdmX on p53 oscillations. Each plot corresponds to the plot in Figure 6-C. In general, similar trends are observed regarding the effects of MdmX in each plot, but the degree of dampening, suppression, or positive effects is reduced in some cases (1, 4, 5, 6) compared to the effects in Figure 6-C. (0.08 MB TIF) [file pcbi.1000665.s002.tif]

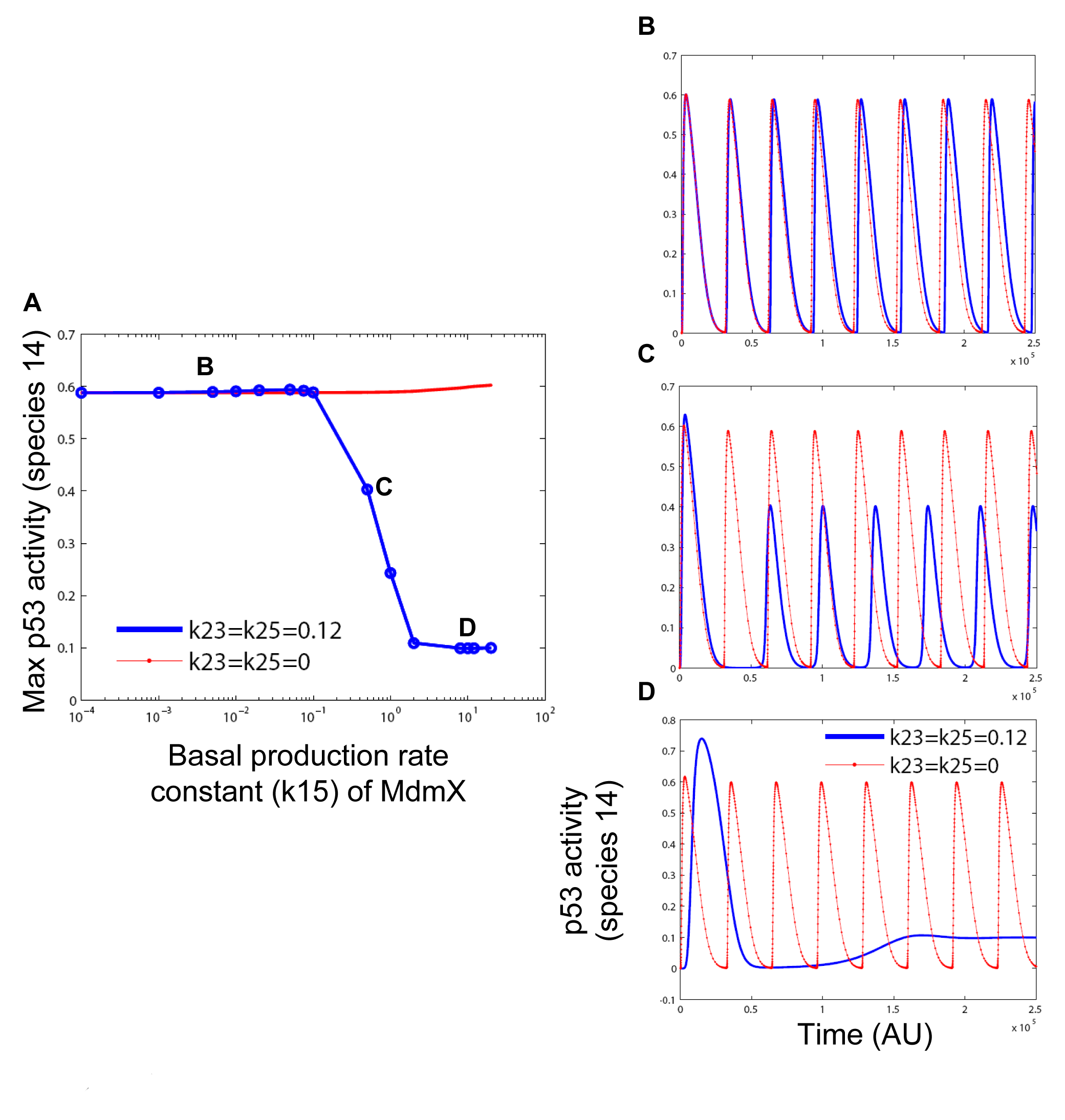

Supplement: Figure S3 — Effects of p53∶MdmX and Mdm2∶MdmX heterodimer reservoirs on p53 oscillatory behavior. The model is based on Figure 2A including the p53-Mdm2 negative feedback loop (k33>0) without time delay and also includes the TR3 mediated positive feedback loop (k14>0). The used kinetic parameter set is listed in Table 1. Simulations were performed with various levels of the basal production rate constant (k15) of MdmX with (k23 = k25 = 0.12) and without (k23 = k25 = 0) heterodimer formation. Maximal (Max) p53 activity was evaluated during the last half (t = 250000 to t = 500000 [AU]) of the simulated period, while the basal production rate constant (k15) of MdmX was varied. (A) p53 activity was estimated with (blue line) and without (red line) formation of p53∶MdmX and Mdm2∶MdmX heterodimers. The p53 activity curve obtained from a model including formation of heterodimers has three characteristic areas indicated as B (no dampening of oscillations), C (reduced oscillatory peaks), and D (dampened oscillations). Time simulation data corresponding to B, C, and D area of curves in (A) are shown in panel (B), (C), and (D) respectively. The dampening effect of MdmX on oscillations was not observed when p53∶MdmX and Mdm2∶MdmX heterodimers were excluded from the model (k23 = k25 = 0). For kinetic constants used, see Table 1. (0.64 MB TIF) [file pcbi.1000665.s003.tif]

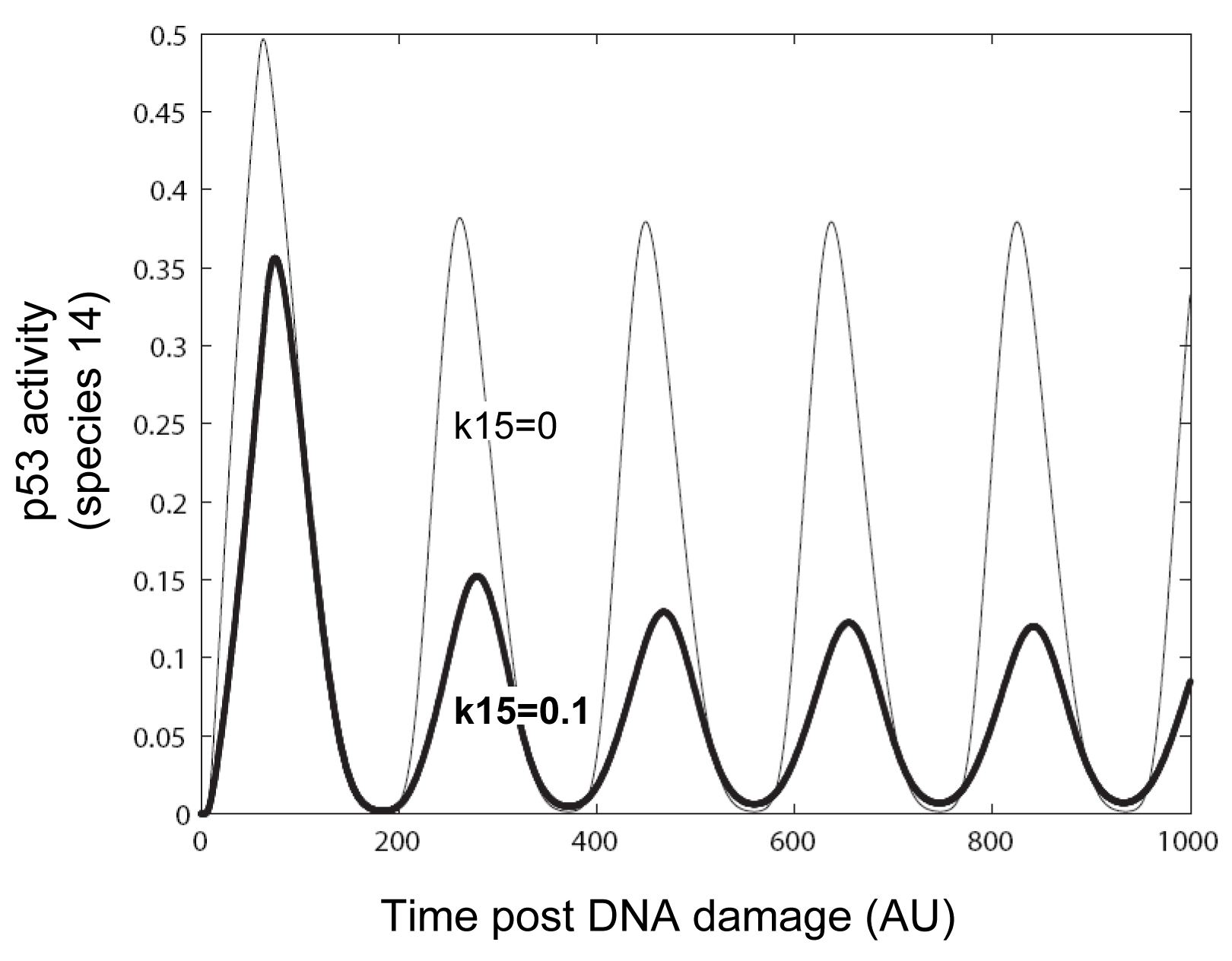

Supplement: Figure S4 — MdmX-induced reduction of the amplitude of oscillating p53. This simulation included the p53-Mdm2 feedback loop (k33>0) with a time delay and without the TR3-mediated positive feedback loop (k14 = 0), and the model is based on Figure 2-A. The thin line represents simulation without MdmX (k15 = 0). The dark line represents simulations with MdmX production rate at k15 = 0.1. For kinetic constants used for simulation, see Table 1. (0.28 MB TIF) [file pcbi.1000665.s004.tif]

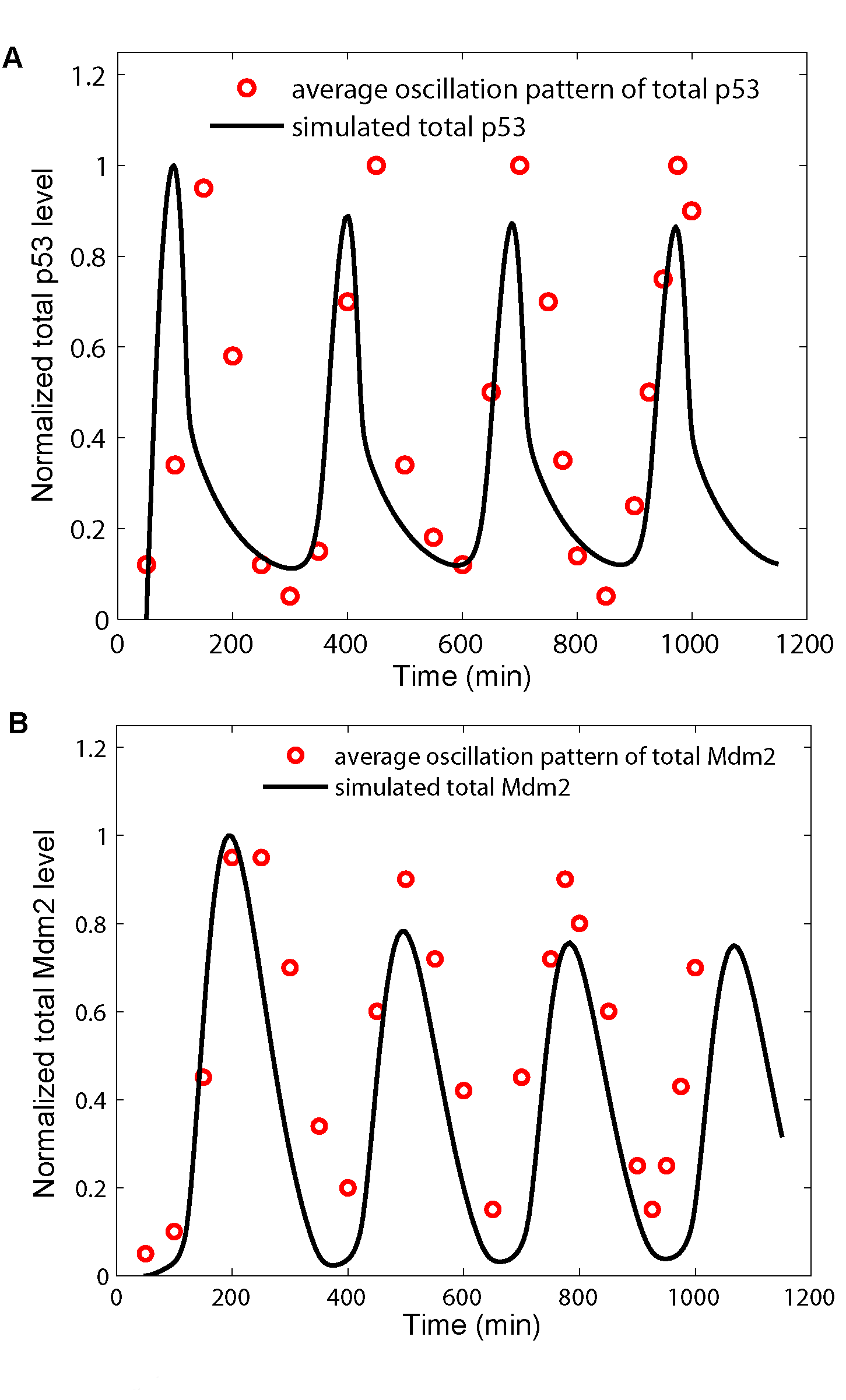

Supplement: Figure S6 — Simulations were performed based on the model in Figure 2B. Simulation results with one set of parameters used for Figure 10 (Table 1) were plot against the averaged oscillatory pattern. To fit to the experimentally observed oscillation period, first, we manually extracted data points from the average oscillation pattern (Fig. S6 in Geva-Zatorski et al. 2006 MSB). Next, we searched for a parameter set which produces approximately similar oscillation interval with the averaged oscillation pattern, and the fit was evaluated by cross-correlation. With the parameter set used in Figure 10, the first peak of total p53 in simulated data appeared and peaked earlier than the averaged p53 oscillation pattern because cross-correlation does not capture whether the signal begins at specific time point or not. Given the observed variability in oscillations (Geva-Zatorski et al. 2006 MSB), we did not intend to fit the exact time point of each peak. The following figures show the overlay of averaged oscillation pattern of each protein and simulated data. For better comparison, the simulated data were plotted by shifting 50 min. For the simulations in the following two figures, same initial condition in Table S2 was used. (0.14 MB TIF) [file pcbi.1000665.s006.tif]

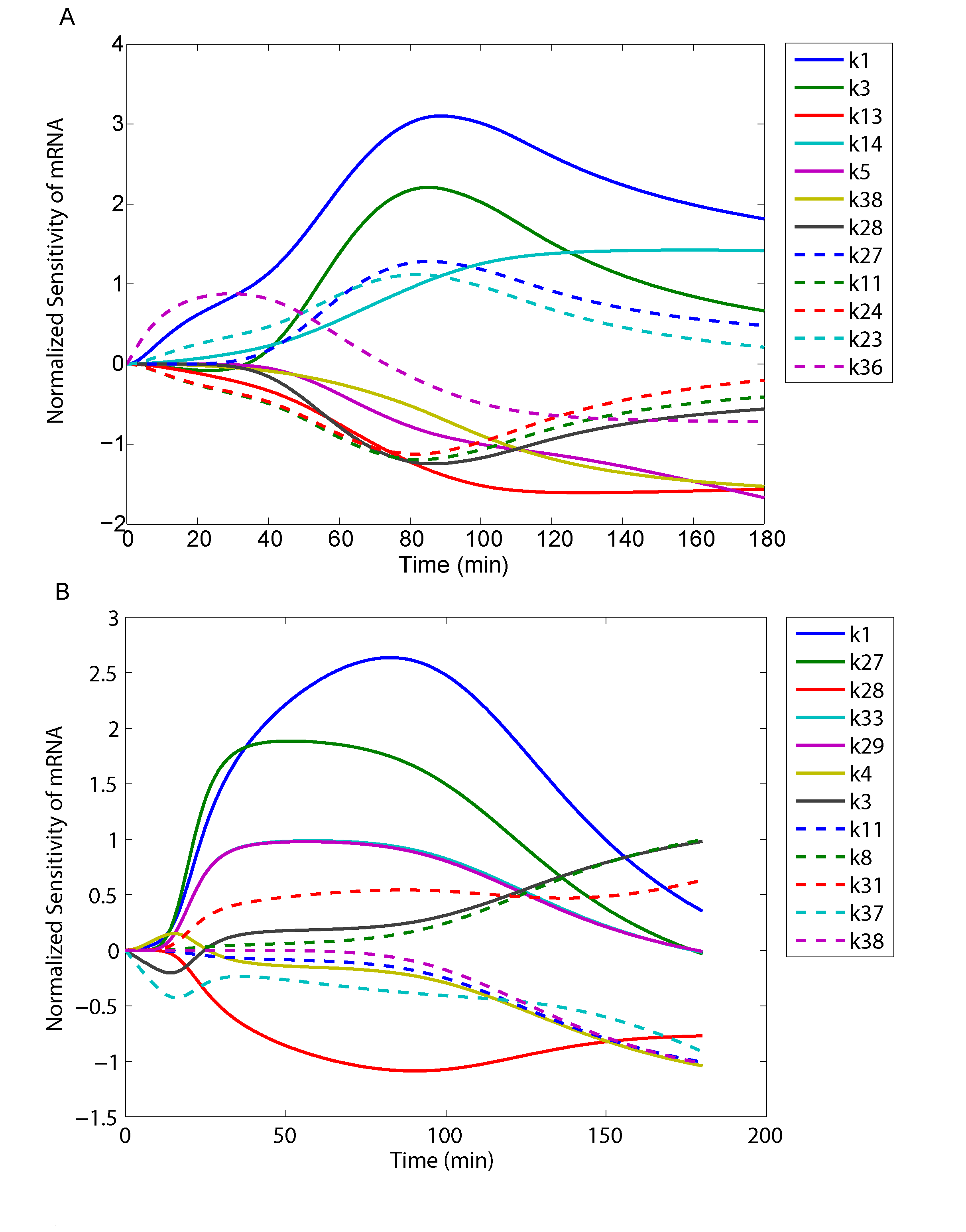

Supplement: Figure S7 — The normalized local sensitivity (LSij) of mRNA (species 15 in Figure 2B) with respect to various parameters. Among 38 kinetic parameters, the 12 most sensitive parameters based on the time integral (see Materials and Methods) were plotted for (A) parameters derived from OSC1 (column 8, Table 1) and (B) parameters derived from OSC2 (column 9, Table 1). (0.21 MB TIF) [file pcbi.1000665.s007.tif]

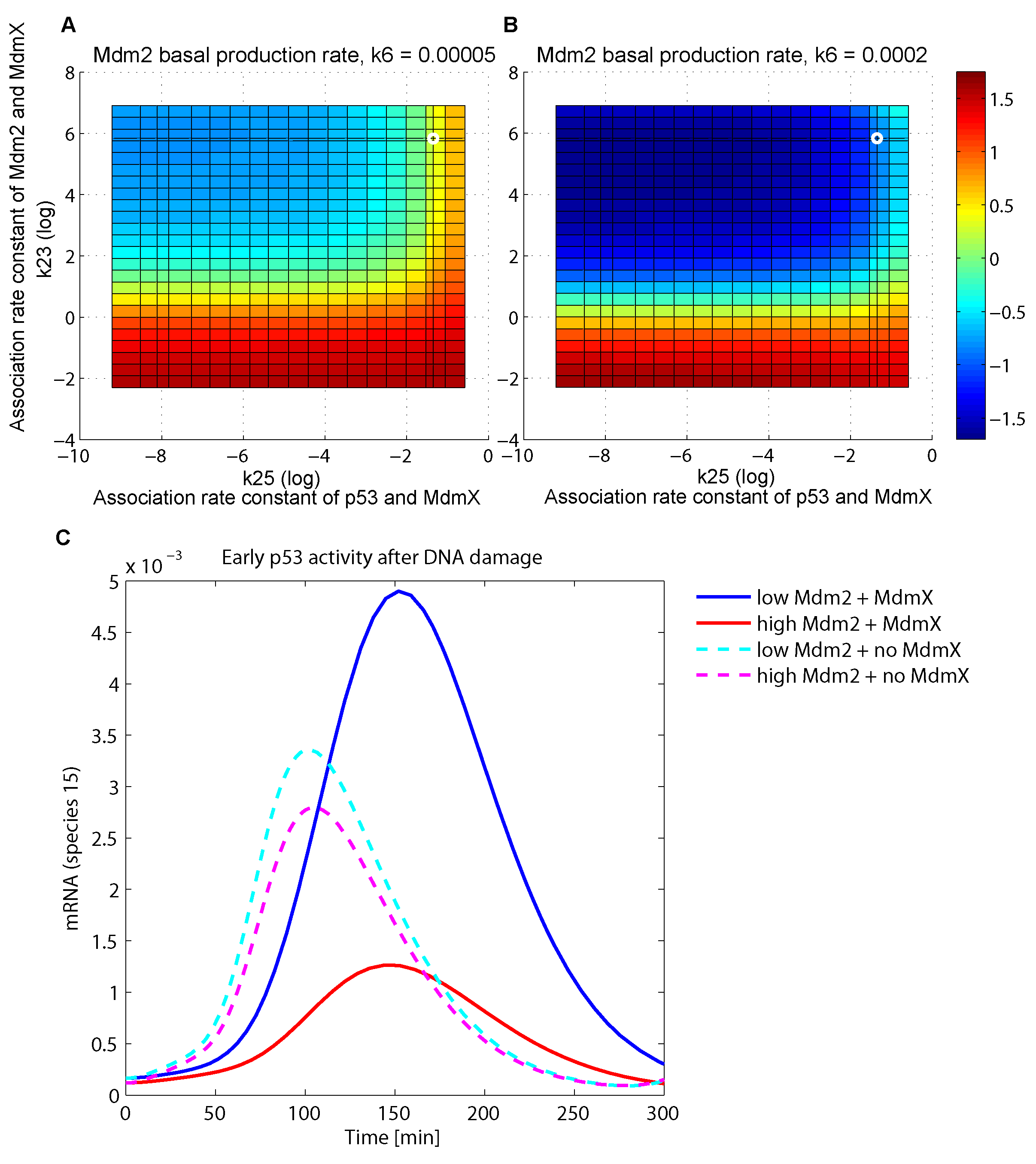

Supplement: Figure S8 — Region of switch-like behaviors as a function of association rate constants of p53∶MdmX and Mdm2∶MdmX. Simulations were performed based on the model in Figure 2-B with pre-equilibration. Log ratio of two p53 activities (with and without MdmX) in Mdm2 = 0.00005 (A) and in Mdm2 = 0.0002 (B) were visualized. Two association rate constants, k23 and k25, were varied from the parameter set used for Figure 9-A (Table 1). The white circle corresponds to the simulation with a parameter set for Figure 9-A. The figure visualizes the log ratio of the p53 activity without MdmX and p53 activity with 100 fold MdmX. The figure shows that when the binding affinity (k25) between p53 and MdmX becomes less tight, the presence of MdmX has negative effect on the p53 activity in a wide range of k23. Also, the figure shows that the switch-like behavior is observed in a wide range of k23 (along the vertical line of white circle) where the yellow (left panel) and blue (right panel) areas overlap. The time series data that correspond to the kinetic parameter sets of white circle (A, B) are shown in panel (C). The time plot shows that the dependency of p53 on Mdm2 increased in the presence of MdmX. (0.26 MB TIF) [file pcbi.1000665.s008.tif]

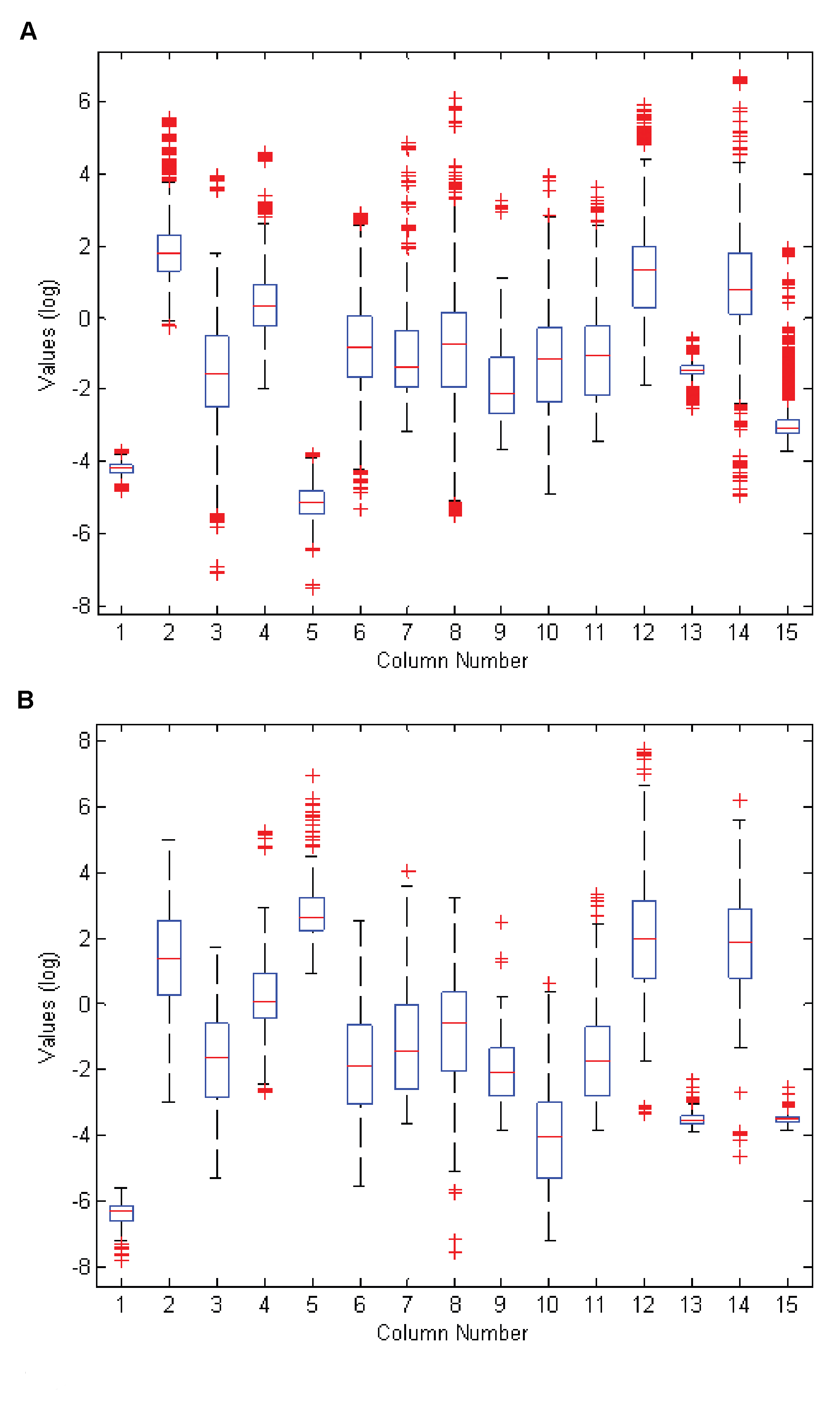

Supplement: Figure S10 — Distribution of each kinetic parameter in cluster OSC1 (A) and OSC2 (B) in Figure 8 (column order: k2, k11, k12, k13, k3, k29, k30, k31, k32, k27, k28, k38, k10, k33, k37). (0.18 MB TIF) [file pcbi.1000665.s010.tif]
