# Supplementary material for: Predicted Functions of MdmX in Fine-Tuning the Response of p53 to DNA Damage
Source: PLoS Comput Biol. 2010 Feb 5;6(2):e1000665. doi: 10.1371/journal.pcbi.1000665 (PMC2824598; doi:10.1371/journal.pcbi.1000665)
Supplement: Table S1 — Annotations of Mdm2-p53-MdmX MIM. (0.13 MB DOC) [file pcbi.1000665.s011.doc]

**Table S1. Annotations** of Mdm2-p53-MdmX MIM

| A1 | P53 can induce transactivation of downstream genes including Mdm2 [1]. |
| --- | --- |
| A2, A3, A4, A5 | The oligomer formation is essential for p53 transactivation function [2] (A2, A3, A4, A5). |
|  | Tetrameric p53 cooperatively binds to DNA. The fundamental active unit of p53 appears to be the tetramer, which is induced by DNA binding, although p53 is a dimer at low concentrations [3] (A3). |
| A6 | Phosphorylation of serine 392 stabilizes the tetramer formation of p53 [4]. |
|  | Based on experiments with protein fragment 303−393, p53 may exist predominantly as monomers in cells with undamaged DNA, because the cellular concentration of p53 is very low in human cells with undamaged DNA (1 to 10 nM) [4]. |
| A7, A15 | Phosphorylation of p53 Ser20 is indirectly mediated by ATM after DNA damage [5] (A15). |
|  | In response to IR and radiomimetic treatment of cells, ATM is activated and mediates rapid phosphorylation of p53 on Ser15 [6]. DNA-PK also phosphorylates p53 at Ser-15 and Ser-37 in vitro [7]. ATR regulates phosphorylation of Ser15 of p53 in DNA-damaged cells [8] (A7). |
|  | In normal lymphoblasts, increased phosphorylation at Ser6, Ser9, Ser15, Ser20, Ser33, Ser46, Ser315, and Ser392 was observed within 15 min after IR [7] (A7, A15). |
|  | Ionizing radiation can induce Ser392-phosphorylation of human p53 [9] (A7). |
| A8, A9, A10, A11, A11-b | Mdm2 ubiquitinates p53 by the Mdm2 ring domain (A10, A11, A11-b), and the ubiquitination of p53 leasds (A9) to the degradation (A8) of p53 [10]. |
| A12, A29, A39 | Hausp deubiquitinates p53 (A12), Mdm2 (A29), and MdmX (A39) [11]. |
| A13 | Oligomerization of p53 is required for p53 to be efficiently ubiquitinated by Mdm2. Deletion mutants that disrupted the oligomerization domain of p53 displayed low binding affinity for MDM2 and were poor substrates for ubiquitination [12]. |
| A14 | Phosphorylation of p53 Ser20 in vivo attenuates the binding of wild-type p53 to Mdm2 [13,14]. |
| A15 | See A7 |
| A16 | Mdm2 residues 16-24 form a lid that closes over the p53-binding site. Modifications to the Mdm2 lid may disrupt p53-Mdm2 binding leading to p53 stabilization [15]. |
| A17 | Phosphorylation of Ser17 Mdm2 by DNA-PK prevents the p53–Mdm2 interaction in vitro [16]. |
|  | Phosphorylation of Mdm2 Ser395 by ATM attenuates the p53-inhibitory potential of Mdm2. Phosphorylation of Mdm2 on ser395 may be less capable of promoting the nucleo-cytoplasmic shuttling of p53 and its subsequent degradation [17]. |
|  | For the phosphorylation of unknown phosphorylation sites, see A16. |
| A18 | MdmX is p53 binding protein [18]. |
| A19, A20 | Phosphorylation of MdmX Ser289 by Casein Kinase 1 alpha (CK1-alpha) (A20) stimulates MdmX-p53 binding (A19). It is unclear whether the enzyme activity of CK1-alpha is regulated by DNA damage in mammalian cells [19]. |
| A21 | Phosphorylation of p53 by DNA-PK leads to a threefold reduction in the interaction between p53 and MdmX. However, phosphorylation of MdmX fails to reduce its interaction with p53 (fig 6) [20]. |
| A22, A23 | Ubiquitin ligase activity of Mdm2 toward Mdm2 was detected in Vitro essay using SF-9 cell extract [21]. |
| A24, A25, A26 | Mdm2 may be active as a dimer or oligomer (A25, A26) as suggested by the threshold of Mdm2 level for autoubiquitination (A24) [22]. |
| A27, A28 | P53 mediates the negative regulation of Mdm2 by orphan regulator TR3. Interaction with p53 is crucial (A28) for TR3 to increase ubiquitinated Mdm2 (A26) [23]. |
| A29 | See A12. |
| A30, A31, A38, A40 | ATM-dependent phosphorylation (A31, A38) lowers affinity of Mdm2 (A30) and MdmX (A40) for the deubiquitinating enzyme HAUSP [11]. |
|  | Daxx enhances the interaction between Hausp and Mdm2. DNA-damage signals disrupt the Mdm2–Daxx–Hausp complex, at least in part, through the activation of ATM. Although it is known that ser395 is phosphorylated by ATM, mutations that either prevent or mimic this phosphorylation exhibited similar binding to Daxx compared with wild-type Mdm2. Therefore, the Mdm2–Daxx–Hausp complex may be regulated by ATM through an additional phosphorylation event [24] (A30, A31). |
| A32, A33, A34, A35, A36 | Mdm2 promotes (A33) MdmX ubiquitination (A34) and degradation (A32) by proteasomes. An intact RING domain of Mdm2 is required (A35), both to interact (A36) with MdmX and to provide E3 ligase function (A34) [25]. |
| A37 | Phosphorylated MdmX binds to Mdm2 with higher affinity [26]. |
| A38 | MdmX ser367 phosphorylation is induced by DNA damage (A38). The enhanced ser367 phosphorylation after DNA damage coincides with increased binding of MdmX to 14-3-3 and accelerated MdmX degradation [27]. |
|  | MdmX ser403 is a direct ATM target. Alanine substitutions at ser403 diminished damage-induced degradation of HdmX [28] (A38). |
|  | See A30. |
| A39 | See A12. |
| A40 | ATM-dependent phosphorylation lowers affinity of Mdm2 and MdmX for the deubiquitinating enzyme HAUSP [11]. |

References:

1. Perry ME (2004) Mdm2 in the response to radiation. Mol Cancer Res 2: 9-19.

2. Kawaguchi T, Kato S, Otsuka K, Watanabe G, Kumabe T, et al. (2005) The relationship among p53 oligomer formation, structure and transcriptional activity using a comprehensive missense mutation library. Oncogene 24: 6976-6981.

3. Weinberg RL, Veprintsev DB, Fersht AR (2004) Cooperative binding of tetrameric p53 to DNA. J Mol Biol 341: 1145-1159.

4. Sakaguchi K, Sakamoto H, Lewis MS, Anderson CW, Erickson JW, et al. (1997) Phosphorylation of serine 392 stabilizes the tetramer formation of tumor suppressor protein p53. Biochemistry 36: 10117-10124.

5. Hirao A, Kong YY, Matsuoka S, Wakeham A, Ruland J, et al. (2000) DNA damage-induced activation of p53 by the checkpoint kinase Chk2. Science 287: 1824-1827.

6. Khosravi R, Maya R, Gottlieb T, Oren M, Shiloh Y, et al. (1999) Rapid ATM-dependent phosphorylation of MDM2 precedes p53 accumulation in response to DNA damage. Proc Natl Acad Sci U S A 96: 14973-14977.

7. Saito S, Goodarzi AA, Higashimoto Y, Noda Y, Lees-Miller SP, et al. (2002) ATM mediates phosphorylation at multiple p53 sites, including Ser(46), in response to ionizing radiation. J Biol Chem 277: 12491-12494.

8. Tibbetts RS, Brumbaugh KM, Williams JM, Sarkaria JN, Cliby WA, et al. (1999) A role for ATR in the DNA damage-induced phosphorylation of p53. Genes and Development 13: 152-157.

9. Blaydes JP, Craig AL, Wallace M, Ball HM, Traynor NJ, et al. (2000) Synergistic activation of p53-dependent transcription by two cooperating damage recognition pathways. Oncogene 19: 3829-3839.

10. Honda R, Tanaka H, Yasuda H (1997) Oncoprotein MDM2 is a ubiquitin ligase E3 for tumor suppressor p53. FEBS Lett 420: 25-27.

11. Meulmeester E, Pereg Y, Shiloh Y, Jochemsen AG (2005) ATM-mediated phosphorylations inhibit Mdmx/Mdm2 stabilization by HAUSP in favor of p53 activation. Cell Cycle 4: 1166-1170.

12. Maki CG (1999) Oligomerization is required for p53 to be efficiently ubiquitinated by MDM2. J Biol Chem 274: 16531-16535.

13. Unger T, Juven-Gershon T, Moallem E, Berger M, Vogt Sionov R, et al. (1999) Critical role for Ser20 of human p53 in the negative regulation of p53 by Mdm2. Embo J 18: 1805-1814.

14. Dumaz N, Milne DM, Jardine LJ, Meek DW (2001) Critical roles for the serine 20, but not the serine 15, phosphorylation site and for the polyproline domain in regulating p53 turnover. Biochem J 359: 459-464.

15. McCoy MA, Gesell JJ, Senior MM, Wyss DF (2003) Flexible lid to the p53-binding domain of human Mdm2: implications for p53 regulation. Proc Natl Acad Sci U S A 100: 1645-1648.

16. Mayo LD, Turchi JJ, Berberich SJ (1997) Mdm-2 phosphorylation by DNA-dependent protein kinase prevents interaction with p53. Cancer Res 57: 5013-5016.

17. Maya R, Balass M, Kim ST, Shkedy D, Leal JF, et al. (2001) ATM-dependent phosphorylation of Mdm2 on serine 395: role in p53 activation by DNA damage. Genes Dev 15: 1067-1077.

18. Shvarts A, Steegenga WT, Riteco N, van Laar T, Dekker P, et al. (1996) MDMX: a novel p53-binding protein with some functional properties of MDM2. Embo J 15: 5349-5357.

19. Chen L, Li C, Pan Y, Chen J (2005) Regulation of p53-MDMX interaction by casein kinase 1 alpha. Mol Cell Biol 25: 6509-6520.

20. Jackson MW, Berberich SJ (1999) Constitutive mdmx expression during cell growth, differentiation, and DNA damage. DNA Cell Biol 18: 693-700.

21. Honda R, Yasuda H (2000) Activity of MDM2, a ubiquitin ligase, toward p53 or itself is dependent on the RING finger domain of the ligase. Oncogene 19: 1473-1476.

22. Linares LK, Hengstermann A, Ciechanover A, Muller S, Scheffner M (2003) HdmX stimulates Hdm2-mediated ubiquitination and degradation of p53. Proc Natl Acad Sci U S A 100: 12009-12014.

23. Zhao BX, Chen HZ, Lei NZ, Li GD, Zhao WX, et al. (2006) p53 mediates the negative regulation of MDM2 by orphan receptor TR3. Embo J 25: 5703-5715.

24. Tang J, Qu LK, Zhang J, Wang W, Michaelson JS, et al. (2006) Critical role for Daxx in regulating Mdm2. Nat Cell Biol 8: 855-862.

25. Pan Y, Chen J (2003) MDM2 promotes ubiquitination and degradation of MDMX. Mol Cell Biol 23: 5113-5121.

26. Chen L, Gilkes DM, Pan Y, Lane WS, Chen J (2005) ATM and Chk2-dependent phosphorylation of MDMX contribute to p53 activation after DNA damage. Embo J 24: 3411-3422.

27. Okamoto K, Kashima K, Pereg Y, Ishida M, Yamazaki S, et al. (2005) DNA Damage-Induced Phosphorylation of MdmX at Serine 367 Activates p53 by Targeting MdmX for Mdm2-Dependent Degradation. Mol Cell Biol 25: 9608-9620.

28. Pereg Y, Shkedy D, de Graaf P, Meulmeester E, Edelson-Averbukh M, et al. (2005) Phosphorylation of Hdmx mediates its Hdm2- and ATM-dependent degradation in response to DNA damage. Proc Natl Acad Sci U S A 102: 5056-5061.
