# Supplementary material for: Predicted Functions of MdmX in Fine-Tuning the Response of p53 to DNA Damage
Source: PLoS Comput Biol. 2010 Feb 5;6(2):e1000665. doi: 10.1371/journal.pcbi.1000665 (PMC2824598; doi:10.1371/journal.pcbi.1000665)
Supplement: Table S2 — Initial condition. (0.04 MB DOC) [file pcbi.1000665.s012.doc]

Table S2. Initial condition

| Symbol | Corresponding species | Initial conditions |
| --- | --- | --- |
| x1 | p53 | 0 |
| x2 | phosphorylated p53 (p53P) | 0 |
| x3 | Mdm2 | 0 |
| x4 | phosphorylated Mdm2 (Mdm2P) | 0 |
| x5 | MdmX | 0 |
| x6 | phosphorylated MdmX (MdmXP) | 0 |
| x7 | p53:Mdm2 heterodimer | 0 |
| x8 | Mdm2:MdmXP heterodimer | 0 |
| x9 | Mdm2:MdmX heterodimer | 0 |
| x10 | p53:MdmX heterodimer | 0 |
| x11 | p53P:p53P homodimer | 0 |
| x12 | (p53P:p53P):(p53P:p53P) tetramer | 0 |
| x13 | Promoter | 1 |
| x14 | ((p53P:p53P): (p53P:p53P)):promoter | 0 |
| x15 | mRNA | 0 |
| x16 | p53:promoter | 0 |
