# Supplementary material for: Predicted Functions of MdmX in Fine-Tuning the Response of p53 to DNA Damage
Source: PLoS Comput Biol. 2010 Feb 5;6(2):e1000665. doi: 10.1371/journal.pcbi.1000665 (PMC2824598; doi:10.1371/journal.pcbi.1000665)
Supplement: Table S3 — Rank ordered time integral (LSj) of the normalized local sensitivity of the kinetic parameter sets from (A) Table 1 column 8 and (B)Table 1 column 9. (0.11 MB DOC) [file pcbi.1000665.s013.doc]

Table S3-A. Rank ordered time integral (*LS_j_*) of the normalized local sensitivity of the kinetic parameter set (Table 1 column 8).

| Rank | LSj | Parameter # | Involved reactions of the parameter |
| --- | --- | --- | --- |
| 1 | 29.009 | k1 | in -> in + p53 |
| 2 | 17.452 | k3 | p53 -> p53p |
| 3 | 16.361 | k13 | [p53:Mdm2] -> Mdm2 |
| 4 | 13.989 | k14 | [p53:Mdm2] -> p53 |
| 5 | 12.77 | k5 | p53p -> null |
| 6 | 12.39 | k38 | mRNA -> Mdm2 + mRNA |
| 7 | 10.517 | k28 | [p53p:p53p] -> p53p + p53p |
| 8 | 10.47 | k27 | p53p + p53p -> [p53p:p53p] |
| 9 | 10.074 | k11 | p53 + Mdm2 -> [p53:Mdm2] |
| 10 | 8.995 | k24 | [Mdm2:MdmX] -> Mdm2 + MdmX |
| 11 | 8.963 | k23 | Mdm2 + MdmX -> [Mdm2:MdmX] |
| 12 | 8.2633 | k36 | [p53:promoter] -> [p53:promoter] + mRNA |
| 13 | 8.2305 | k34 | p53 + promoter -> [p53:promoter] |
| 14 | 8.1911 | k35 | [p53:promoter] -> p53 + promoter |
| 15 | 6.7377 | k30 | [p53p:p53p:p53p:p53p] -> [p53p:p53p] + [p53p:p53p] |
| 16 | 6.359 | k32 | [p53p:p53p:p53p:p53p:promoter] -> [p53p:p53p:p53p:p53p] + promoter |
| 17 | 5.8854 | k33 | [p53p:p53p:p53p:p53p:promoter] -> [p53p:p53p:p53p:p53p:promoter] + mRNA |
| 18 | 5.8613 | k31 | [p53p:p53p:p53p:p53p] + promoter -> [p53p:p53p:p53p:p53p:promoter] |
| 19 | 5.6685 | k29 | [p53p:p53p] + [p53p:p53p] -> [p53p:p53p:p53p:p53p] |
| 20 | 5.6567 | k17 | MdmX -> MdmXp |
| 21 | 5.1621 | k37 | mRNA -> null |
| 22 | 1.4437 | k12 | [p53:Mdm2] -> p53 + Mdm2 |
| 23 | 1.1673 | k2 | p53 -> null |
| 24 | 1.1215 | k25 | p53 + MdmX -> [p53:MdmX] |
| 25 | 1.1158 | k26 | [p53:MdmX] -> p53 + MdmX |
| 26 | 0.67295 | k6 | in -> in + Mdm2 |
| 27 | 0.51002 | k8 | Mdm2 -> Mdm2p |
| 28 | 0.28516 | k4 | p53p -> p53 |
| 29 | 0.15446 | k15 | in -> in + MdmX |
| 30 | 0.12211 | k22 | [Mdm2:MdmXp] -> Mdm2 |
| 31 | 0.11549 | k16 | MdmX -> null |
| 32 | 0.068572 | k20 | Mdm2 + MdmXp -> [Mdm2:MdmXp] |
| 33 | 0.019565 | k18 | MdmXp -> MdmX |
| 34 | 0.011414 | k21 | [Mdm2:MdmXp] -> Mdm2 + MdmXp |
| 35 | 0.002458 | k7 | Mdm2 -> null |
| 36 | 0.001958 | k19 | MdmXp -> null |
| 37 | 0.000764 | k9 | Mdm2p -> Mdm2 |
| 38 | 0.000671 | k10 | Mdm2p -> null |

^a^$LS_{j}=\sqrt{\int_{t=0}^{t=T} \left[\left(\frac{k_{j}}{x_{15}(t)}\right)\left(\frac{\partial x_{15}(t)}{\partial k_{j}}\right)\right]^{2}dt},T=180$

Table S3-B. Rank ordered time integral (*LS_j_*) of the normalized local sensitivity of the kinetic parameter set (Table 1 column 9).

| Rank | *LSj*^a^ | Parameter # | Involved reactions of the parameter |
| --- | --- | --- | --- |
| 1 | 24.184 | k1 | in -> in + p53 |
| 2 | 17.127 | k27 | p53p + p53p -> [p53p:p53p] |
| 3 | 11.332 | k 28 | [p53p:p53p] -> p53p + p53p |
| 4 | 9.1343 | k33 | [p53p:p53p:p53p:p53p:promoter] -> [p53p:p53p:p53p:p53p:promoter] + mRNA |
| 5 | 9.0018 | k29 | [p53p:p53p] + [p53p:p53p] -> [p53p:p53p:p53p:p53p] |
| 6 | 6.7316 | k4 | mRNA -> null |
| 7 | 6.6134 | k3 | [p53p:p53p:p53p:p53p] + promoter -> [p53p:p53p:p53p:p53p:promoter] |
| 8 | 6.4257 | k11 | [p53p:p53p:p53p:p53p:promoter] -> [p53p:p53p:p53p:p53p] + promoter |
| 9 | 6.3242 | k8 | [p53p:p53p:p53p:p53p] -> [p53p:p53p] + [p53p:p53p] |
| 10 | 6.2908 | k31 | p53p -> p53 |
| 11 | 6.2325 | k37 | p53 + Mdm2 -> [p53:Mdm2] |
| 12 | 6.2173 | k38 | p53 -> p53p |
| 13 | 6.0517 | k32 | Mdm2 -> Mdm2p |
| 14 | 6.0339 | k30 | mRNA -> Mdm2 + mRNA |
| 15 | 4.7021 | k9 | Mdm2p -> Mdm2 |
| 16 | 1.7191 | k5 | p53p -> null |
| 17 | 1.5968 | k10 | [p53:promoter] -> [p53:promoter] + mRNA |
| 18 | 1.2746 | k13 | [p53:promoter] -> p53 + promoter |
| 19 | 1.0417 | k14 | p53 + promoter -> [p53:promoter] |
| 20 | 0.98571 | k36 | Mdm2p -> null |
| 21 | 0.98508 | k35 | [p53:Mdm2] -> Mdm2 |
| 22 | 0.98473 | k34 | [p53:MdmX] -> p53 + MdmX |
| 23 | 0.85717 | k26 | [p53:Mdm2] -> p53 |
| 24 | 0.23779 | k12 | p53 + MdmX -> [p53:MdmX] |
| 25 | 0.21189 | k25 | MdmX -> MdmXp |
| 26 | 0.21017 | k17 | [p53:Mdm2] -> p53 + Mdm2 |
| 27 | 0.1728 | k18 | MdmXp -> MdmX |
| 28 | 0.16614 | k20 | Mdm2 + MdmXp -> [Mdm2:MdmXp] |
| 29 | 0.090212 | k2 | p53 -> null |
| 30 | 0.030131 | k7 | [Mdm2:MdmX] -> Mdm2 + MdmX |
| 31 | 0.028563 | k24 | in -> in + Mdm2 |
| 32 | 0.020314 | k6 | Mdm2 -> null |
| 33 | 0.007353 | k21 | [Mdm2:MdmXp] -> Mdm2 + MdmXp |
| 34 | 0.0055 | k22 | [Mdm2:MdmXp] -> Mdm2 |
| 35 | 0.003655 | k23 | Mdm2 + MdmX -> [Mdm2:MdmX] |
| 36 | 0.000461 | k15 | in -> in + MdmX |
| 37 | 0.000408 | k19 | MdmXp -> null |
| 38 | 1.18E-05 | k16 | MdmX -> null |

^a^$LS_{j}=\sqrt{\int_{t=0}^{t=T} \left[\left(\frac{k_{j}}{x_{15}(t)}\right)\left(\frac{\partial x_{15}(t)}{\partial k_{j}}\right)\right]^{2}dt},T=180$
