# Supplementary material for: Predicted Functions of MdmX in Fine-Tuning the Response of p53 to DNA Damage
Source: PLoS Comput Biol. 2010 Feb 5;6(2):e1000665. doi: 10.1371/journal.pcbi.1000665 (PMC2824598; doi:10.1371/journal.pcbi.1000665)
Supplement: Table S4 — Ordinary differential equations for figure 2-B (symbols are in Table 1). (0.03 MB DOC) [file pcbi.1000665.s014.doc]

Table S4. Ordinary differential equations for figure 2-B (symbols are in Table 1)

| d[x1]/dt=k1-k2*[x1]-k3*[x1]+k4*[x2]-k11*[x1]*[x3]+k12*[x7]+k14*[x7]-k25*[x1]*[x5]+k26*[x10]-k34*[x1]*[x13]+k35*[x16] |
| --- |
| d[x2]/dt=k3*[x1]-k4*[x2]-k5*[x2]-2*k27*[x2]*[x2]+2*k28*[x11] |
| d[x3]/dt=k6-k7*[x3]-k8*[x3]+k9*[x4]-k11*[x1]*[x3]+k12*[x7]+k13*[x7]-k20*[x3]*[x6]+k21*[x8]+k22*[x8]-k23*[x3]*[x5]+k24*[x9]+k38*[x15] |
| d[x4]/dt=k8*[x3]-k9*[x4]-k10*[x4] |
| d[x5]/dt=k15-k16*[x5]-k17*[x5]+k18*[x6]-k23*[x3]*[x5]+k24*[x9]-k25*[x1]*[x5]+k26*[x10] |
| d[x6]/dt=k17*[x5]-k18*[x6]-k19*[x6]-k20*[x3]*[x6]+k21*[x8] |
| d[x7]/dt=k11*[x1]*[x3]-k12*[x7]-k13*[x7]-k14*[x7] |
| d[x8]/dt=k20*[x3]*[x6]-k21*[x8]-k22*[x8] |
| d[x9]/dt=k23*[x3]*[x5]-k24*[x9] |
| d[x10]/dt=k25*[x1]*[x5]-k26*[x10] |
| d[x11]/dt=k27*[x2]*[x2]-k28*[x11]-2*k29*[x11]*[x11] +2*k30*[x12] |
| d[x12]/dt=k29*[x11]*[x11]-k30*[x12]-k31*[x12]*[x13]+k32*[x14] |
| d[x13]/dt=-k31*[x12]*[x13]+k32*[x14]-k34*[x1]*[x13]+k35*[x16] |
| d[x14]/dt=k31*[x12]*[x13]-k32*[x14] |
| d[x15]/dt=k33*[x14]-k37*[x15]+k36*[x16] |
| d[x16]/dt=k34*[x1]*[x13]-k35*[x16] |
