# Supplementary material for: Predicted Functions of MdmX in Fine-Tuning the Response of p53 to DNA Damage
Source: PLoS Comput Biol. 2010 Feb 5;6(2):e1000665. doi: 10.1371/journal.pcbi.1000665 (PMC2824598; doi:10.1371/journal.pcbi.1000665)
Supplement: Table S5 — Kinetic constants used in previous mathematical models. (0.04 MB DOC) [file pcbi.1000665.s015.doc]

Table S5. Kinetic constants used in previous mathematical models

|  | By Ciliberto *et al.* [1] | By Ma *et al.* [2] | By Bar-Or *et al.*[3] | Values in the initial parameter set | corresponding symbols in our model |
| --- | --- | --- | --- | --- | --- |
| Basal induction of mdm2 | 0.0015 /min | 0.0018 uM/min | 0.00235 | 0.0015 | k6 |
| Increased induction of mdm2 by p53 | max: 0.006 /min | txn: 0.024 uM/min tln: 0.02 /min | max: ~0.03 | 0.01 | k33 |
| Basal degradation of mdm2 | 0.01 /min | 0.0028 /min | 0.05 | 0.005 | k7 |
| Increased degradation of mdm2 given DNA damage | 0.02 /min | 0.014 /min | NA | 20 | k10 |
| phosphorylation of mdm2 | max: 0.05 /min | NA | NA | 0.02 | k8 |
| dephospohrylation of mdm2 | 6 /min | NA | NA | 0.02 | k9 |
| basal production of p53 | 0.055 /min | 0.02 uM/min | 0.5 | 0.055 | k1 |
| basal degradation of p53 | 0.0055 /min | 0.0184~0.02 /min | 0.00025 | 0.0055 | k2 |
| mdm2 dependent degradation of p53 | NA | 0.092 /min | NA | 5 | k13 |
| mdm2 dependent degradation of UU-p53 | 0.0055 ~ 8 /min | NA |

NA indicates that corresponding kinetic parameter values were not available.

**Reference:**

1. Ciliberto A, Novak B, Tyson JJ (2005) Steady states and oscillations in the p53/Mdm2 network. Cell Cycle 4: 488-493.

2. Ma L, Wagner J, Rice JJ, Hu W, Levine AJ, et al. (2005) A plausible model for the digital response of p53 to DNA damage. Proc Natl Acad Sci U S A 102: 14266-14271.

3. Lev Bar-Or R, Maya R, Segel LA, Alon U, Levine AJ, et al. (2000) Generation of oscillations by the p53-Mdm2 feedback loop: a theoretical and experimental study. Proc Natl Acad Sci U S A 97: 11250-11255.
