# Supplementary material for: Predicted Functions of MdmX in Fine-Tuning the Response of p53 to DNA Damage
Source: PLoS Comput Biol. 2010 Feb 5;6(2):e1000665. doi: 10.1371/journal.pcbi.1000665 (PMC2824598; doi:10.1371/journal.pcbi.1000665)
Supplement: Table S6 — Assumptions used to generate an initial kinetic parameter set (column 3 in Table 1). (0.03 MB DOC) [file pcbi.1000665.s016.doc]

Table S6. Assumptions used to generate an initial kinetic parameter set (column 3 in Table 1)

| Assumptions | Description |
| --- | --- |
| k11=k23=k25 > k12=k24=k26 | Binding affinity of heterodimerization of unphosphorylated molecules is similar^a^. |
| k27=k29=k31 > k28=k30=k32 | Association and dissociation constants are similar for oligomerization of p53 and promoter binding ^a^. Association constants are larger than dissociation constants ^a^. |
| k2 > k7=k16=k19 | Basal degradation of Mdm2, MdmX, and phosphorylated MdmX are similarly processed^a^. p53 undergoes more active basal degradation than Mdm2, MdmX, and unphosphorylated MdmX^a^. |
| k2 >= k5 | p53 may undergo more active basal degradation than unphosphorylated p53^a^. |
| k3=k8=k17 | Each molecule undergoes phosphorylation with similar rate ^a^, and the rate remains constant [1]. |
| k4=k9=k18 | Each phosphorylated molecule can be similarly dephosphorylated (similar level of dephosphorylation activity) ^a^. |
| Large k10, k13, k22 | Mdm2 dependant degradation of p53 (degradation by ubiquitinaiton) and phosphorylated MdmX are quite rapid [2]. Degradation of phosphorylated Mdm2 is also quite rapid (A30 in Figure 1) [3]. |
| k10 >> k7 | Phosphorylated Mdm2 undergoes active degradation than unphophorylated Mdm2 (A30 in Figure 1). |
| k20 >> k21 | MdmX is rapidly removed by Mdm2 after DNA damage (A33, A38, A40). |

^a^The condition was assumed for simplicity.

1. Bakkenist CJ, Kastan MB (2003) DNA damage activates ATM through intermolecular autophosphorylation and dimer dissociation. Nature 421: 499-506.

2. Pan Y, Chen J (2003) MDM2 promotes ubiquitination and degradation of MDMX. Mol Cell Biol 23: 5113-5121.

3. Meulmeester E, Pereg Y, Shiloh Y, Jochemsen AG (2005) ATM-mediated phosphorylations inhibit Mdmx/Mdm2 stabilization by HAUSP in favor of p53 activation. Cell Cycle 4: 1166-1170.
